# Scalar-Flux Similarity in the Layer Near the Surface Over Mountainous Terrain

**DOI:** 10.1007/s10546-018-0365-3

**Published:** 2018-06-14

**Authors:** Eleni Sfyri, Mathias W. Rotach, Ivana Stiperski, Fred C. Bosveld, Manuela Lehner, Friedrich Obleitner

**Affiliations:** 1 Department of Atmospheric and Cryospheric Sciences, University of Innsbruck, Innrain 52f, 6020 Innsbruck, Austria; 2 Royal Netherlands Meteorological Institute, 3701 AE De Bilt, The Netherlands

**Keywords:** Complex terrain, Horizontally inhomogeneous, I-Box experimental site, Local similarity

## Abstract

The scaled standard deviations of temperature and humidity are investigated in complex terrain. The study area is a steep Alpine valley, with six measurement sites of different slope, orientation and roughness (i-Box experimental site, Inn Valley, Austria). Examined here are several assumptions forming the basis of Monin–Obukhov similarity theory (MOST), including constant turbulence fluxes with height and the degree of self-correlation between the involved turbulence variables. Since the basic assumptions for the applicability of the MOST approach—horizontally homogeneous and flat conditions—are violated, the analysis is performed based on a local similarity hypothesis. The scaled standard deviations as a function of local stability are compared with previous studies from horizontally homogeneous and flat terrain, horizontally inhomogeneous and flat terrain, weakly inhomogeneous and flat terrain, as well as complex terrain. As a reference, similarity relations for unstable and stable conditions are evaluated using turbulence data from the weakly inhomogeneous and flat terrain of the Cabauw experimental site in the Netherlands, and assessed with the same post-processing method as the i-Box data. Significant differences from the reference curve and also among the i-Box sites are noted, especially for data derived from the i-Box sites with steep slopes. These differences concern the slope and the magnitude of the best-fit curves, illustrating the site dependence of any similarity theory.

## Introduction

In recent years, a growing number of studies have focused on turbulence structure in truly complex and mountainous terrain (e.g., Rotach et al. 2004; Moraes et al. 2005; Rotach and Zardi 2007; Fernando et al. 2015; Stiperski and Rotach 2016). The understanding of boundary-layer processes in complex terrain is crucial in atmospheric modelling, e.g., for numerical weather prediction, climate, air pollution and hydrosphere–cryosphere modelling (de Bruin et al. 1993; Baklanov et al. 2011). Improved boundary-layer parametrizations and turbulence closure strategies for high-resolution models are needed for applications that take into account the complexity of the terrain and the flow conditions, as well as canopy flows. Although turbulent exchange processes in complex topography have been investigated over the last few decades (e.g.,Rotach and Zardi 2007; Fernando et al. 2015), relatively little work (an exception being Nadeau et al. 2013a, b) has been devoted to the systematic investigation of scaling relations in complex terrain. Therefore, in practical applications, scaling relations developed over horizontally homogeneous and flat (HHF) terrain are often employed.

Turbulent fluxes can be directly measured using the eddy-covariance method, which is based on the covariance between turbulent fluctuations of scalars and the velocity vector (Aubinet et al. 2012). However, as data are not always available from routine meteorological observations, such as in operational networks, similarity theory is a useful tool, since only a few non-dimensional parameters provided by basic measurements are needed to estimate the main turbulence variables (e.g.,Holtslag and Nieuwstadt 1986; Stull 1988). The early focus of similarity scaling was based mainly on ideal conditions, as well as horizontally homogeneous and flat terrain, constant fluxes with height in the surface layer, and quasi-stationary turbulence with very small uncertainties.

To compare turbulence characteristics between study areas in different conditions, a proper scaling method is needed. The most usual and widely-accepted similarity scaling for the atmospheric surface layer is Monin–Obukhov similarity theory (MOST) (Monin and Obukhov 1954), in which the scaled turbulence variances of the flow depend only on the stability *z* / *L*, where *z* is the measurement height, and *L* is the Obukhov length, and is an appropriate theory over HHF terrain and under ideal conditions. Under these conditions, the MOST functions of the scaled turbulence variables are considered to be universal. We note that the MOST approach is based on the hypothesis that the surface fluxes are in equilibrium with the local fluxes, so the surface fluxes are used to define the scaling parameters at any measurement height within the surface layer. Even if originally derived for the surface layer, MOST cannot be applied to the entire surface layer, but it is only valid in its upper part, i.e., the inertial sublayer (e.g.,Rotach and Calanca 2015). Furthermore, for strongly unstable conditions, the standard deviations of the horizontal velocity components ($\sigma _u$, $\sigma _v$) are influenced by large eddies extending throughout the unstable boundary layer, so that these variables do not obey surface-layer scaling (Panofsky et al. 1977; Holtslag and Nieuwstadt 1986).

Therefore, the above criteria are not universally met, and it is questionable whether the universal functions apply in the case of inhomogeneous terrain. As an alternative, the applicability of local similarity has been investigated. The parameters of the similarity functions are assumed to be ‘localized’, i.e. specific for the area (site) under consideration. Generally, the original form of these scaling relations has not changed, except for the respective coefficients (e.g.,Andreas et al. 1998; Ramana et al. 2004; Moraes et al. 2005; Nadeau et al. 2013a).

If MOST is not applicable, a useful approach is local scaling for which turbulent quantities are a function of $z/\varLambda$, where $\varLambda$ is the local Obukhov length, but is a much less powerful framework than MOST. For instance, the surface fluxes cannot be retrieved from local scaling if there are no clearly verified characteristics of the fluxes (as will be demonstrated in Sect. 5). Nevertheless, if the assumptions for MOST are not fulfilled, knowledge of the surface fluxes may be of less importance, and local scaling can be considered as a first step towards a better understanding of turbulence characteristics in complex terrain.

After establishing the theoretical framework (Sect. 2) and introduction of the dataset (Sect. 3), the reference parametrizations employed are introduced in Sect. 4. Our objective is to investigate the applicability of similarity theory for normalized standard deviations of scalars in a complex Alpine valley. The crucial conditions that allow the application of Monin–Obukhov similarity theory are evaluated in Sect. 5, and local similarity theory is investigated for temperature and humidity standard deviations in Sect. 6.

## Theoretical Background

### Similarity Scaling

According to the MOST approach, each dimensionless variable of the flow can be described as a function of only the measurement height *z*, the surface sensible heat and momentum fluxes, and the buoyancy parameter (Wyngaard et al. 1971), with the latter three variables represented by the Obukhov length *L*. For local scaling, *L* is replaced by the local Obukhov length,

1 $$\begin{aligned} \varLambda = -\frac{1}{\kappa }\frac{u_{*l}^3}{\overline{w'\theta '}_l}\left( \frac{g}{{\overline{\theta }}}\right) ^{-1}, \end{aligned}$$

where the subscript *l* denotes local, $u_{*l}$ is the local friction velocity, *u*, *v*, *w* are the streamwise, spanwise and slope-normal velocity components, $\theta$ is the virtual potential air temperature, $\overline{(w'\theta ')}_l$ is the local buoyancy flux, $\kappa$ is the von Kármán constant ($\kappa = 0.4$) and *g* is the acceleration due to gravity. The friction velocity is given by Kaimal and Finnigan (1994) as

2 $$\begin{aligned} u_{*l} = \left( {\overline{u'w'}_l^2+\overline{v'w'}_l^2}\right) ^{1/4}. \end{aligned}$$

The ratio $\zeta = z/\varLambda$ is defined as the local stability parameter, indicating the stability at the height of the measurement. The velocity variables are normalized by the friction velocity (2), while the temperature variables are normalized by a characteristic temperature scale $\theta _{*l}$,

3 $$\begin{aligned} \theta _{*l} = -\frac{\overline{w'\theta '}_l}{u_{*l}}, \end{aligned}$$

and the humidity variables are normalized by the characteristic humidity scale $q_{*l}$,

4 $$\begin{aligned} q_{*l} = -\frac{\overline{w'q'}_l}{u_{*l}}. \end{aligned}$$

Therefore, the scaled standard deviation of temperature and humidity is $\varPhi _{\theta } = \sigma _{\theta }/|\theta _{*l}|$ and $\varPhi _q = \sigma _q/q_{*l}$, respectively. For $\varPhi _{\theta }$, we use the absolute value $|\theta _{*l}|$ for normalization, so as to represent both the stable and unstable relationships on logarithmic axes.

Many studies have dealt with the applicability of similarity scaling of $\varPhi _{\theta }$ and $\varPhi _q$ in various types of terrain (Tables 1, 2), with the majority—and in particular those referring to HHF terrain—using a MOST framework where stability is characterized by *z* / *L* rather than $z/\varLambda$. To be consistent with the foregoing (and the presentation of our own results), we will use the local Obukhov length (1) and, hence, $z/\varLambda$ as the stability parameter hereafter (including Tables  1, 2), even if the MOST framework and correspondingly *z* / *L* was used previously. The general form of the similarity functions for unstable ($z/\varLambda \le 0$) and stable ($z/\varLambda \ge 0$) stratification can be written as

5 $$\begin{aligned} \varPhi _i = a_i(b_i + c_iz/\varLambda )^{d_i} + e_i, \end{aligned}$$

where $\varPhi _{i}$ is the scaled standard deviation of the variable *i* ($i = \theta$ or *q*), and $a_i$, $b_i$, $c_i$, $d_i$ and $e_i$ are the appropriate similarity coefficients.

The majority of previous studies propose a similarity function that is only valid outside the near-neutral limit $|z/\varLambda | > 0.05$ (e.g., Moraes et al. 2005; Nadeau et al. 2013a); otherwise, $\varPhi _{\theta }$ shows significant variability for unstable conditions in the case of non-HHF terrain. In neutral conditions over HHF terrain, the vertical heat flux tends towards zero and the temperature fluctuations become very small, so that the scaled temperature variance tends to be finite (Monin and Yaglom 1971). However, in non-ideal conditions, such as in complex terrain, the temperature fluctuations remain finite (Tampieri et al. 2009), even as the heat flux goes to zero when approaching neutral conditions, thus causing the scaled temperature variance to diverge. For this reason, we treat the near-neutral (unstable) region, with the more strongly unstable ranges of $z/\varLambda$ treated separately for $\varPhi _{\theta }$. Recently, Tampieri et al. (2009) suggested that, in the near-neutral (unstable) region ($- 0.05\le z/\varLambda \le 0$), the scaling relation for the temperature standard deviation as a function of $z/\varLambda$ has an exponent $d_{\theta } = - 1$, rather than the classical $d_{\theta } = - 1/3$. On the stable side, many of the early studies suggested a constant $\varPhi _{\theta }$ (e.g., Nieuwstadt 1984; Liu et al. 1998. However, Pahlow et al. (2001) recommended an exponent of $- 1$ for near-neutral (stable) conditions in horizontally inhomogeneous and flat terrain. As for the humidity, the variability of $\varPhi _q$ in the near-neutral range has not been reported in the literature, because the humidity flux $\overline{w'q'}$ does not tend to zero when approaching neutral stratification.

### Terrain Influence on Turbulence: An Overview

Summarized in Tables 1 and 2 are the similarity functions for scaled temperature and humidity standard deviations as proposed in various turbulence studies for different types of terrain. The range of stability parameter $\left| z/\varLambda \right|$, in which every similarity function is valid, depends on the available data range and on the definition of the near-neutral limit (usually $\left| z/\varLambda \right| = 0.01 - 0.05$). The different types of terrain are classified herein as follows: horizontally homogeneous and flat (HHF); horizontally inhomogeneous and flat (HIF); weakly inhomogeneous and flat (WIF) and complex terrain. The WIF terrain type is used only for the Cabauw study area as explained in Sect. 4 (Beljaars and Bosveld 1997; Bosveld 1999). Here, weakly inhomogeneous refers to terrain with very low roughness elements on the order of 0.1 m or less in height, or with taller roughness elements far from the measurement site.

**Fig. 1:**
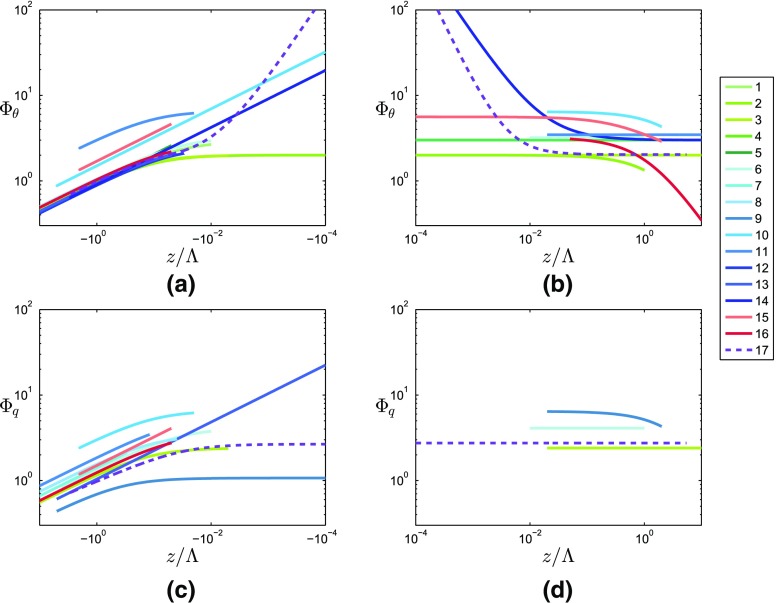
Existing similarity functions of non-dimensional temperature (**a**, **b**) and humidity (**c**, **d**) standard deviations as a function of local stability, under unstable (left) and stable (right) conditions. The numbers in the legend refer to the references listed in Tables 1 and 2. The curves used as a reference are based on the Cabauw dataset, and are shown as purple dashed lines and are highlighted. Note that, for consistency, all curves are expressed in a local scaling framework (even if the original publication used a MOST framework)

The majority of the similarity functions for $\varPhi _{\theta }$($z/\varLambda$) found in the literature have the same $d_{\theta } = -1/3$ exponent in unstable conditions, but different coefficients $a_{\theta }$, $b_{\theta }$, $c_{\theta }$ and $e_{\theta }$. Similarly, studies over complex terrain find the same slope of the curve as the HHF studies for unstable conditions, although the values of $\varPhi _{\theta }$ differ (see Fig. 1a). On the stable side, several studies suggest that $\varPhi _{\theta }$ is independent of $z/\varLambda$ (zero exponent) (e.g., Shao and Hacker 1990; Liu et al. 1998; Andreas et al. 1998), others suggest an exponent of $- 1$ (e.g., Kaimal and Finnigan 1994; Pahlow et al. 2001; Ramana et al. 2004; Nadeau et al. 2013a) or an exponent of $- 1/3$ (e.g., Quan and Hu 2009; Moraes et al. 2005). According to these functions found in the literature, no clear connection between the exponent of $\varPhi _{\theta }$ and the terrain type is apparent.

The similarity relation between the scaled standard deviation of humidity $\varPhi _q$ and $z/\varLambda$ has always been a matter of controversy among different studies, especially on the stable side, probably because of the potential influence of small evaporation or condensation fluxes. As shown in Table 2, a limited number of studies address $\varPhi _q$ for unstable conditions (e.g., Nadeau et al. 2013a), but very few for stable conditions, possibly because of the large scatter for stable stratification and the large uncertainty of humidity-fluctuation measurements. Several studies suggest that humidity behaves similarly to temperature, so they use the same expression (e.g., Ramana et al. 2004). In contrast, Andreas et al. (1998) and Liu et al. (1998) suggest that $\varPhi _q$ does not depend on $z/\varLambda$.

Figure 1 shows the similarity functions listed in Tables 1 and 2. In Fig. 12 (see Appendix), the same information is displayed separately for the four different terrain types. The numbering of the curves corresponds to the numbers of the different functions in Tables 1 and 2, and as shown, the curves differ in magnitude, slope and range of validity. It can be seen in Fig. 12a–d that the blue (HIF terrain) and the red (complex terrain) curves exhibit large variability unlike those for HHF terrain (green), for which curves are more similar. The fact that the curves for HHF terrain are similar but not identical leads us to propose a new reference curve based on data from the meteorological tower in Cabauw, the Netherlands (see Sect. 4), whose reference curves are depicted by purple dashed lines in Figs. 1 and  12.

**Table 1:** List of similarity relations for the non-dimensional temperature standard deviation found in the literature

A/A	References	Terrain	Unstable					Stable					Stability range
			$a_{\theta }$	$b_{\theta }$	$c_{\theta }$	$d_{\theta }$	$e_{\theta }$	$a_{\theta }$	$b_{\theta }$	$c_{\theta }$	$d_{\theta }$	$e_{\theta }$	
1	de Bruin et al. (1993)	HHF	2.9	1	− 28.4	− 1/3	0						− 100 $\le$ $z/\varLambda \le -0.01$
2	Kaimal and Finnigan (1994)	HHF	2	1	− 9.5	− 1/3	0						− 2$\le$ $z/\varLambda \le$ 0
								2	1	0.5	− 1	0	0 $\le$ $z/\varLambda \le$ 1
3	Liu et al. (1998)	HHF	2	1	− 8	− 1/3	0						$- 40 \le z/\varLambda < -5\times 10^{-3}$
								–	–	–	–	2	0.02$\le z/\varLambda<$ 10
4	Nieuwstadt (1984)	HHF						–	–	–	–	3	$z/\varLambda>$ 0
5	Wyngaard et al. (1971)	HHF	0.95	0	$- 1$	$- 1/3$	0						$- 2.5< z/\varLambda < -0.05$
6	Andreas et al. (1998)	HIF	3.2	1	$- 28.4$	$- 1/3$	0						$- 4 < z/\varLambda \le -0.01$
								–	–	–	–	3.2	0.01 $\le z/\varLambda<$ 1
7	Asanuma and Brutsaert (1998)	HIF	0.92	0	$-1$	$- 1/3$	0						$-40$ $\le z/\varLambda < -0.3$
8	Cava et al. (2008)	HIF	2.3	1	$-9.5$	$- 1/3$	0						$- 20 \le z/\varLambda \le -0.04$
9	Pahlow et al. (2001)	HIF						0.05	0	1	$-1$	3	$4\times 10^{-3} \le z/\varLambda \le$ 40
10	Quan and Hu (2009)	HIF	1.5	0	$-1$	$-1/3$	0						$- 5< z/\varLambda<$ 0
								3	0	1	$$- $$ 1/3	0	0 $$< z/\varLambda<$$ 5
11	Ramana et al. (2004)	HIF	6.56	1	$-9.5$	− 1/3	0						$- 2 < z/\varLambda \le -0.02$
								6.45	1	0.25	− 1	0	0.02 $< z/\varLambda \le$ 2
12	Shao and Hacker (1990)	HIF	$\sqrt{6}$	1	$- 20$	− 1/3	0						$- 80 < z/\varLambda \le -0.03$
								–	–	–	–	$\sqrt{12}$	0.04 $\le z/\varLambda \le$ 30
13	Tillman (1972)	HIF	0.95	0.055	− 1	− 1/3	0						$- 60 < z/\varLambda \le -0.05$
14	Högstrom and Smedman (1974)	HIF	0.91	0	− 1	− 1/3	0						$-3 < z/\varLambda \le$ 0
15	Moraes et al. (2005)	Complex	1.7	0	− 1	− 1/3	0						$- 2 < z/\varLambda \le -0.05$
								5.6	1	3.1	− 1/3	0	0 $< z/\varLambda<$ 2
16	Nadeau et al. (2013a)	Complex	2.67	1	− 16.29	− 1/3	0						$z/\varLambda < -0.05$
								3.22	1	0.83	− 1	0	$z/\varLambda>$ 0.05
17	Reference curve	WIF	0.99	0.063	− 1	− 1/3	0						$-2.87\le z/\varLambda \le -0.05$
			0.015	0	− 1	− 1	1.76						$-0.05< z/\varLambda \le -7\times 10^{-5}$
								$8.7\times 10^{-4}$	0	1	− 1.4	2.03	$1.4\times 10^{-5} \le z/\varLambda \le 5.5$

The coefficients $a_{\theta }$, $b_{\theta }$, $c_{\theta }$, $d_{\theta }$, $e_{\theta }$ refer to the general formula (5)

**Table 2:** List of similarity relations for the non-dimensional humidity standard deviation as found in the literature

A/A	References	Terrain	Unstable					Stable					Stability range
			$a_q$	$b_q$	$c_q$	$d_q$	$e_q$	$a_q$	$b_q$	$c_q$	$d_q$	$e_q$	
3	Liu et al. (1998)	HHF	2.4	1	− 8	− 1/3	0						$-40\le z/\varLambda < -0.005$
								–	–	–	–	2.4	0.02$\le z/\varLambda \le$ 4
6	Andreas et al. (1998)	HIF	4.1	1	− 28.4	− 1/3	0						$-4 < z/\varLambda \le -0.01$
								–	–	–	–	4.1	0.01 $\le z/\varLambda < 1$
7	Asanuma and Brutsaert (1998)	HIF	1.42	0	− 1	− 1/3	0						$-40 \le z/\varLambda < -0.3$
8	Cava et al. (2008)	HIF	3.4	1	− 9.5	− 1/3	0						$-20\le z/\varLambda \le -0.04$
10	Quan and Hu (2009)	HIF	1.07	1	− 2.71	− 1/3	0						$-5< z/\varLambda < 0$
11	Ramana et al. (2004)	HIF	6.56	1	− 9.5	− 1/3	0						$-2 < z/\varLambda \le -0.02$
								6.45	1	0.25	− 1	0	0.02 $< z/\varLambda \le$ 2
12	Shao and Hacker (1990)	HIF	$\sqrt{30}$	1	− 25	− 1/3	0						$-80 < z/\varLambda \le -0.12$
14	Högstrom and Smedman (1974)	HIF	1.04	0	− 1	− 1/3	0						$-5 \le z/\varLambda<$ 0
15	Moraes et al. (2005)	Complex	1.5	0	− 1	− 1/3	0						$-2 < z/\varLambda \le -0.05$
16	Nadeau et al. (2013a)	Complex	3.51	1	− 21.74	− 1/3	0						$z/\varLambda < -0.05$
17	Reference curve	WIF	0.99	0.031	− 1	− 0.288	0						$-2.87 \le z/\varLambda \le -7\times 10^{-5}$
								–	–	–	–	2.74	$1.4\times 10^{-5} \le z/\varLambda \le 5.5$

The coefficients $a_q$, $b_q$, $c_q$, $d_q$, $e_q$ refer to the general formula (5)

## Data and Methods

### Datasets

**Fig. 2:**
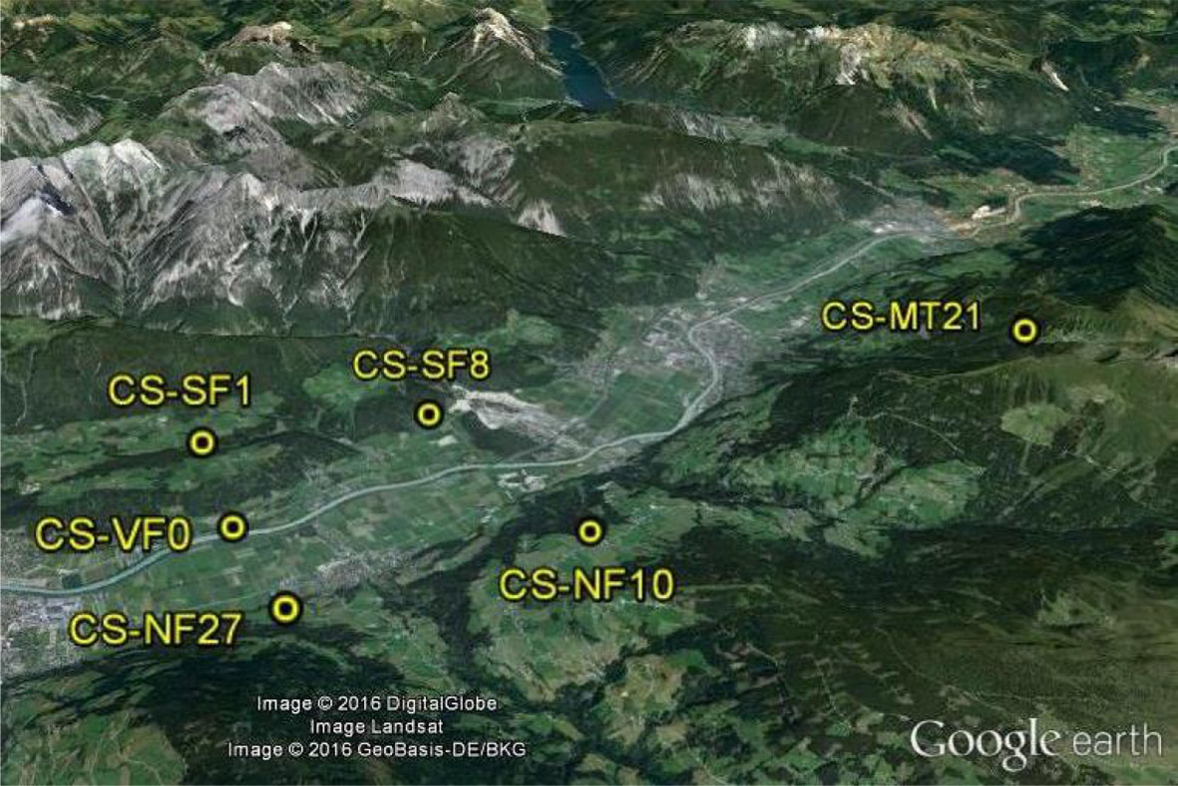
The i-Box sites in the Inn Valley in Austria (taken from Google Earth)

Data are obtained from five measurement sites in an Alpine valley (Inn Valley, Austria) with different slope angles, orientation and vegetation (see Table 3, Fig. 2). The study area is referred to as i-Box, and provides a framework for studying boundary-layer processes in complex terrain (Rotach et al. 2017). The i-Box site is located in a valley with very complex terrain, oriented from approximately north-east to south-west. The locations of the i-Box sites were chosen in such a way to include representative topographic characteristics of the valley: valley floor, north–south orientation, mountain top, different vegetation and terrain slopes (Stiperski and Rotach 2016).

**Table 3:** Main characteristics of the i-Box and Cabauw measurement sites

Station name	Identification	Local slope ($^{\circ }$)	Height of levels (m)	Vegetation	Orientation
Kolsass	CS-VF0	0	4 / 8.7 / 16.9	Mixed agricultural	Valley floor
Terfens	CS-SF8	8	6.2 / 11.3	Agricultural, car-parking	South-facing
Eggen	CS-SF1	1	6.6	Alpine meadow, corn field	South-facing
Weerberg	CS-NF10	10	6.2	Alpine meadow	North-facing
Hochhauser	CS-NF27	27	6.2	Alpine meadow	North-facing
Reference site (Cabauw)	–	0	3	Agricultural, field	Extended plain

The numbers in the identification denote the terrain slope ($^{\circ }$) at every site

*CS* core site, *VF* valley floor, *SF* south facing, *NF* north facing

The list of sites used here is given in Table 3, where the names indicate the orientation and the terrain slope in degrees for every site. Three sites have one level, while the CS-SF8 and CS-VF0 sites have two and three levels, respectively. The turbulence measurements at the Cabauw site, which are used as a reference, are made at one measurement level (see also Sect. 4).

At the i-Box sites, velocity components, air and soil temperature, humidity, and the components of the energy balance have been measured since 2013. Used here are turbulence data from CSAT3 sonic anemometers, KH20 Krypton hygrometers, and EC150 open-path gas analyzers (all from Campbell Scientific, Logan, Utah, USA) recorded at a frequency of 20 Hz, covering a period of 2.5 years (August 2013–December 2015). Necessary raw data for the current analysis include the sonic temperature and humidity fluctuations, as well as the velocity components. Low-frequency (1-min) data of air temperature, relative humidity and pressure are used mainly for the purpose of flux corrections. Detailed information about the i-Box sites and the instruments can be found at https://wiki.uibk.ac.at/display/IBOX/i-Box+Home. The aerodynamic roughness characteristics differ between the i-Box sites and depend on the wind direction. The land use of every measurement site is shown in Table 3. For the i-Box sites with only grass (CS-NF27 and CS-NF10), and for the sites with grass and corn (CS-SF1, CS-VF0, CS-SF8), non-linear functions are used to describe the height of the vegetation as a function of the day of the year (i.e. the growing period).

The CS-VF0 site is located between agricultural fields with different vegetation. Using sporadic measurements of the vegetation height *h* through the years 2014 and 2015, the zero-plane displacement *d* was first calculated from the relation $d = 0.7h$, for $30^{\circ }$ sectors of wind direction, taking the different types of fields into consideration, which were included in every sector. By weighted averaging (dependent on the width of every field), one value of *d* was determined for each of the fields, for every wind-direction sector, and for both stable and unstable stratifications. The footprint model of Kljun et al. (2015) was then applied three times using the value from the previous iteration as an initial value for *d*, until the calculated footprint became constant. With these calculated values of zero-plane displacement for every wind-direction sector, a logarithmic best-fit function (following the growth rate of the plants) between *d* and the day of the year was determined separately for stable and unstable conditions. Finally, the calculated zero-plane displacements were subtracted from the measurement height *z*. The calculated best-fit functions were also used for the year 2013, for which no vegetation height for the individual fields had been assessed.

Another correction of the measurement height that may be considered relevant in the i-Box environment is related to snow cover in winter. However, as the snow depth usually does not exceed the typical grass height (when data are available), corresponding to a zero-plane displacement of a few millimetres, snow cover has no discernible influence on our target variables (i.e., $z/\varLambda$) and is, therefore, not taken into account.

For the remaining sites, the method for determining *d* was simpler, because there are only two types of surface cover (grass and corn). Therefore, for these sites, the footprint model of Kljun et al. (2015) was applied to find the fields of maximum influence. Afterwards, non-linear functions between *d* and the day of the year were created for stable and unstable stratifications and for the wind-direction sectors that included grass or corn fields.

**Fig. 3:**
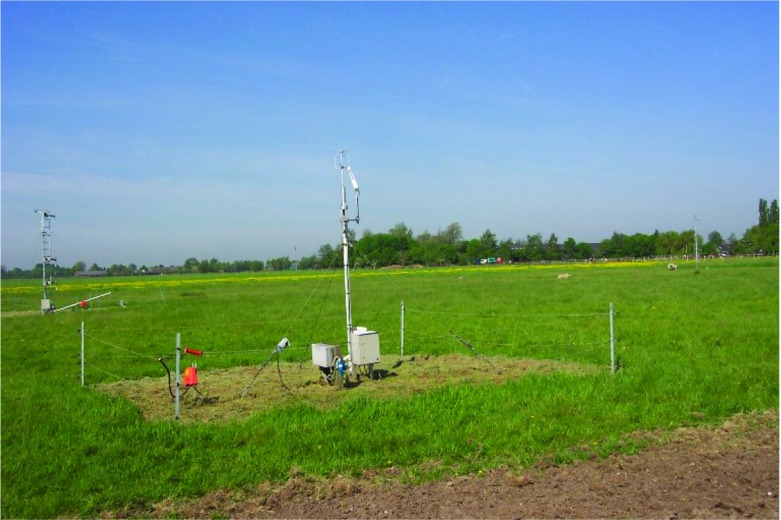
The Cabauw study area in the Netherlands (photo credit: Fred Bosveld)

Figure 3 shows the Cabauw experimental site for atmospheric research (CESAR) of the Royal Netherlands Meteorological Institute (KNMI). The study area is located on a flat and almost horizontally homogeneous field, at a distance of 1 km from a suburban area (see also Sect. 4). The data are obtained from a 3-m turbulence tower and not from the 213-m main tower of the Cabauw site.

### Methods

The 20-Hz raw data from the i-Box sites were processed by the software EdiRe (Version 1.4.3.1101, from R. Clement, University of Edinburgh, UK). We used a recursive filter with a time scale of 200 s to eliminate the low-frequency motions, before calculating 30-min averages of the turbulence parameters. The double-rotation method (Aubinet et al. 2012; Wilczak et al. 2001) was used to rotate the data into a slope-normal streamwise coordinate system. The procedure for ascertaining the highest quality of the data suggested by Stiperski and Rotach (2016) was followed, which includes flux corrections (Schotanus et al. 1983; Moore 1986; Webb et al. 1980), an assessment of the data uncertainty (Wyngaard 1973), a test for stationarity (Foken and Wichura 1996), skewness and kurtosis thresholds for the temperature and velocity components (Vickers and Mahrt 1997), as well as despiking. Here, we used the same threshold values for the quality criteria as Stiperski and Rotach (2016) for their ‘high-quality’ dataset. Following this procedure, we only examine the high-quality 30-min averaged data for use in the following turbulence analysis. The test for high quality reduced the dataset to about 30$\%$ of its original amount, which may raise the question about the generality of the results. In this respect, it should be noted that a considerable fraction of the ‘lost’ data is due to non-stationarity and statistical uncertainty, which are data-quality requirements generally applied even over ideal terrain. A useful comparison can, therefore, only be made when applying these tests also over complex terrain. Furthermore, an a posteriori assessment of the results with relaxed quality criteria (not shown) revealed that none of the results of this study changed significantly—even if the scatter using the larger but less reliable database increased. It is, therefore, argued that—despite the largely reduced amount of data through quality control—the overall characteristics of the obtained results reflect all possible flow conditions, but should nevertheless be employed with caution.

Prior to the quality control, the turbulence data are corrected for unwanted spikes using the following despiking method as applied to the raw data by the EdiRe software; if the difference between a data point and its neighbours is larger than 10 standard deviations, the data point is replaced by the interpolated value derived from the neighbouring data points. This process is applied once for the temperature and velocity components and four times for the humidity fluctuations because the Krypton hygrometers are very sensitive to precipitation, producing many periods with spikes after and during precipitation events.

Additional quality control applied to the humidity data reduces the i-Box dataset size by about 15$\%$, including the rejection of 30-min averages with a relative humidity larger than 95$\%$, as well as data with a minimum voltage of the Krypton hygrometer lower than an instrument-specific threshold (of about 70 mV). Periods with a voltage below this threshold are related to precipitation events and condensation on the instruments, resulting in periodic spikes not removed by the despiking algorithm, as they are systematic.

## Weakly Inhomogeneous and Flat Terrain as Reference

Since our aim is to evaluate to what degree the scalar standard deviations in complex terrain agree with local scaling, there is a need for a reference. The reference is not derived from the HHF similarity functions, because, as shown in Fig. 1, even the HHF similarity functions exhibit some variability. Therefore, it was decided to establish new reference similarity functions based on data from a flat and only marginally horizontally inhomogeneous site, i.e., the Cabauw site in the Netherlands.

For this reason, 11 months of high-frequency data (10 Hz) are used from the database of the Cabauw Experimental Site for Atmospheric Research (CESAR), which includes turbulence measurements from a 3-m mast at the Cabauw field (51.97201$^\circ$N, 4.924847$^\circ$E) recorded with a LI-COR 7500 open-path gas analyzer (LI-COR, Lincoln, Nebraska, USA) and a Gill-R3 sonic anemometer (Gill Instruments, Lymington, Hampshire, UK). The area around the tower is flat with grass fields, and the roughness length is on average 0.03 m. The surrounding terrain is characterized as flat and relatively homogeneous up to a distance of 10 km (Nieuwstadt 1984).

As we compare our results from complex terrain with the reference, and therefore need to be sure that potential differences are not due to post-processing options or the length of the averaging interval, the raw data from Cabauw (reference data) were post-processed with exactly the same post-processing as the i-Box data. Moreover, the high-quality control process consistent with the i-Box data was followed, except for the quality flags from the instruments, which reduced the reference dataset to 51$\%$ of its initial size.

The reason for classifying the Cabauw study area as weakly inhomogeneous and not horizontally homogeneous is that, although the whole area is flat, there are some roughness elements near the measurement site (trees, houses and water canals), but at least 120 m from the measurement tower. Given the low measurement height (3 m) and the comparably large distance to the obstacles, this inhomogeneity does not likely affect the fluxes. Still, to test this assumption rigorously, we applied the footprint model of Kljun et al. (2015). Climatological footprints and several individual footprints were calculated for unstable, stable and near-neutral (slightly unstable or stable) conditions. The source area of the climatological, as well as of the individual, footprints used in the model, is the 80$\%$ impact area. The climatological footprints are the aggregation of the footprints of all the unstable, stable and near-neutral cases, respectively, for the 11-month period. This analysis shows that all the calculated footprint areas do not include the closest roughness elements. However, in some cases with stable stratification, the 80$\%$ footprint area includes several of the nearby water canals, which is the reason we use the term weakly inhomogeneous and flat for the Cabauw study area.

**Fig. 4:**
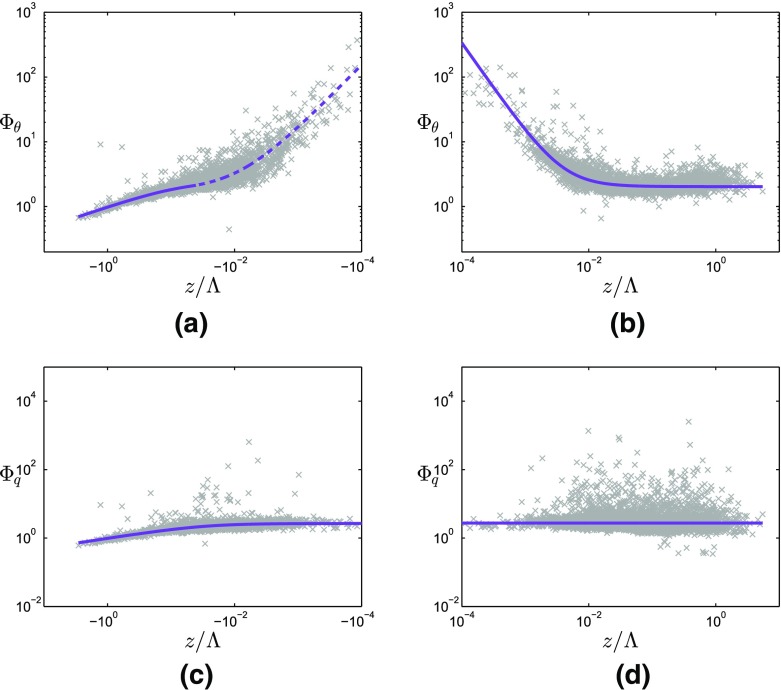
Non-dimensional temperature (**a**, **b**) and humidity (**c**, **d**) standard deviations as a function of local stability for unstable (**a**, **c**) and stable (**b**, **d**) stratification for the reference dataset. Grey symbols: 30-min averages that have passed the high-quality criteria (Sect. 4) from an 11-month dataset at the reference site in Cabauw. Purple lines: best-fit curves to the datasets, which are used as reference curves. Solid line in **a**: formulation suggested by Tillman (1972), dashed line in **a**: formulation suggested by Tampieri et al. (2009). In the fitting procedure, the two curves are blended at $z/\varLambda = -\,0.05$ requiring the same value and derivative

In Fig. 4, $\varPhi _{\theta }$ and $\varPhi _q$ for the reference dataset are plotted as a function of $z/\varLambda$ for unstable and stable stratifications, with the best-fit curves through these data points shown in Tables 1 and 2. To reduce the influence of outliers on the curve fit, the non-linear robust best-fit method with weighting (bisquare) was used. On the unstable side, the best-fit curve was found by fitting 5 to the reference data, using $c_{\theta } = -1$ and $e_{\theta } = 0$, in agreement with other studies (e.g., Tillman 1972; Nadeau et al. 2013a). As the slope of $- 1$ suggested by Tampieri et al. (2009) in the near-neutral (unstable) region of the reference data fits satisfactorily to the data points, their suggested function is also used in this region, i.e. $\varPhi _{\theta } = a_{\theta }(-z/\varLambda )^{-1}+e_{\theta }$.

In Fig. 1, the present reference best-fit curves can be compared with those from the literature. Outside the near-neutral range, both the temperature and humidity reference curves are in the range of those from the literature for the HHF terrain (green lines in Fig. 1). For near-neutral stability, the slope of $\varPhi _{\theta }$ deviates strongly from previous results, with even steeper slopes than reported for the HIF terrain (blue lines). The near-neutral (unstable) slope of $\varPhi _q$ is close to one of the slopes found over HIF terrain, but the magnitude of the reference $\varPhi _q$ does deviate strongly from any of the curves found in the literature.

## Constant-Flux Hypothesis and Self-Correlation Assessment

### Variation of Turbulent Fluxes with Height

The MOST approach can be applied in the upper part (inertial sublayer) of the surface layer where the turbulent fluxes are close to being constant with height (Monin and Obukhov 1954; Nadeau et al. 2013a). According to Mahrt (1999), every study that evaluates the applicability of MOST should include an examination of the constant-flux approximation, since the MOST approach is based on the assumption that the turbulence characteristics at every level depend only on the surface stability parameter. According to Nieuwstadt (1984), local scaling should be used instead of surface-layer scaling when the heat flux and stress change significantly with height. For this reason, it is crucial to evaluate the constant-flux approximation. Even in ideal conditions, the fluxes (especially of momentum) are not expected to be perfectly constant with height. Therefore, by using the term constant-flux layer, we essentially refer to a ‘near-constant’ flux layer.

As shown in Table 3, two i-Box sites have more than one level: the CS-VF0 and CS-SF8 sites. The evaluation of the variability of fluxes with height is mainly done at the CS-VF0 site, as it has three levels, and is confirmed at the CS-SF8 site with two levels. For brevity, we use the term ‘temperature flux’ for the kinematic turbulent flux of sensible heat ($\overline{w'\theta '}$) and the term ‘humidity flux’ for the kinematic turbulent flux of latent heat ($\overline{w'q'}$). The variability of momentum, temperature and humidity fluxes with height is investigated. If the constant-flux hypothesis is not valid on the valley floor (CS-VF0), it is not expected to hold at the other sloped sites either.

Traditionally, to consider a constant flux with height requires in practice that the difference in fluxes should be $\le 10\%$ within the surface layer (e.g., Kaimal and Businger 1970). Here, due to the complexity of the terrain, we used less strict criteria, by considering a change in magnitude of $< 20\%$ between any two measurement levels as ‘approximately’ constant. However, small fluxes with the same sign are also considered to be constant with height. According to Klipp and Mahrt (2004), as small fluxes are often characterized by large random errors, and are affected by mesoscale trends, they are excluded from the constant-flux test. Temperature and humidity fluxes are considered to be small if their absolute values are $< 0.01$ K m s$^{-1}$ and $10^{-5}$ kg m$^{-2}$ s$^{-1}$, respectively. For momentum fluxes, this threshold is set to 0.01 m$^2$ s$^{-2}$.

Even with these less stringent criteria, the percentage of constant fluxes is still very small. Specifically, the percentage of simultaneously constant momentum and temperature fluxes is about $1\%$ for both stable and unstable conditions, whereas the percentage of simultaneously constant momentum and humidity fluxes is about $0.5\%$ for both unstable and stable conditions. The percentage of constant temperature flux is about $50\%$ for both unstable and stable conditions, and for the humidity flux, the percentages are substantially smaller, especially in unstable stratification. Concerning the momentum fluxes, only about $1\%$ are constant, which shows that the low number of simultaneously constant momentum and temperature or humidity fluxes is mainly due to the non-constancy of the momentum fluxes. Therefore, the hypothesis of a near-constant-flux layer does not hold, and local scaling is preferable in the case of complex terrain.

**Fig. 5:**
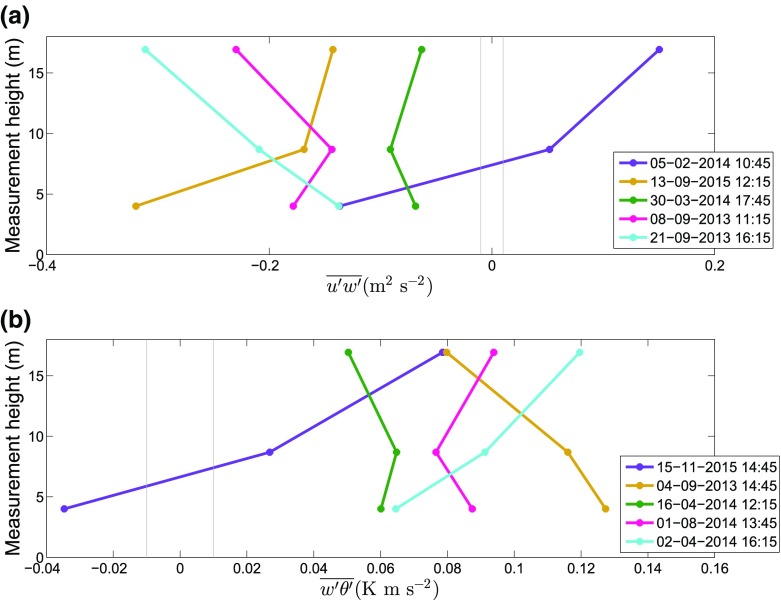
Examples of non-constant fluxes of momentum (**a**), and temperature (**b**) at the CS-VF0 site. Purple: fluxes with different signs, light blue: increasing fluxes with height, yellow: decreasing fluxes with height, green: largest flux at the second level, pink: smallest flux at the second level. The vertical lines on both sides of zero indicate the range of ‘small fluxes’ for which constancy is not checked

**Fig. 6:**
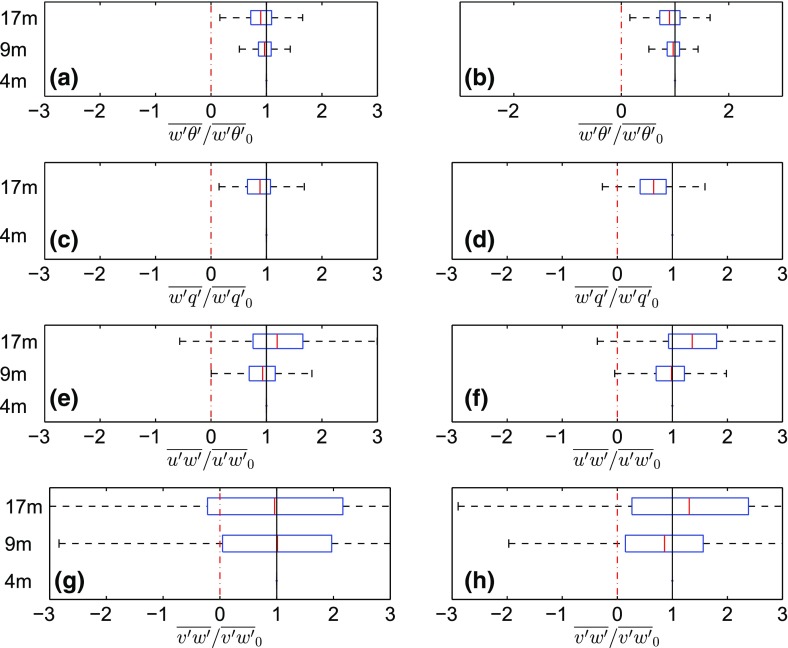
Box plots of temperature (**a**, **b**), humidity (**c**, **d**) and momentum (**e**, **f**, **g**, **h**) fluxes, for the three levels at the CS-VF0 site, for unstable (left) and stable stratification (right). The fluxes are normalized by the corresponding flux at the first level. Black vertical line: constant fluxes, red solid line: median. Both cases of ‘constant’ and ‘non-constant fluxes’ (see text for criteria and abundance) are taken into account

In Fig. 5, examples for the range of possible variations of temperature and momentum fluxes with height are shown for the CS-VF0 site. At sites such as the i-Box sites, i.e. in complex (mountainous) terrain, turbulent fluxes are found to exhibit quite different vertical profiles compared with HHF terrain, i.e. the fluxes vary strongly between the measurement levels. In agreement with Nadeau et al. (2013a), we identified five types of non-constant fluxes: monotonically decreasing or increasing, with relative extrema (minimum or maximum) at the middle level (8.7 m), and fluxes with different signs at different levels. Furthermore, in Fig. 6, we present box plots of the normalized turbulent fluxes for the CS-VF0 site, where it can be seen that, even on average, the turbulent fluxes vary substantially with height. For both stable and unstable conditions, the non-constant temperature and humidity fluxes mostly decrease with height, whereas the momentum fluxes tend to have a minimum at the second level. The small percentage of constant momentum-flux cases is mainly due to the large variability at the top level (Fig. 6). Longitudinal ($\overline{u'w'}$) and lateral ($\overline{v'w'}$) momentum fluxes are on average ‘approximately constant’ over the first two measurement levels, while the third level appears to be situated in a relatively frequent shear zone. However, investigation of the characteristics of this behaviour is beyond the scope here, but will be addressed elsewhere. The directional shear ($\overline{v'w'}$) exhibits a significant case-to-case variability. As the above suggests a lack of a constant-flux layer in the complex environment of the i-Box sites, the MOST approach cannot be employed for scaling.

### Assessment of Self-Correlation

Self-correlation occurs when the dependent and the independent variables in a functional relationship (as in Eq. 5) contain a common variable (Baas et al. 2006). For $\varPhi _{\theta }$ and $\varPhi _q$ as a function of $z/\varLambda$, this common variable is $u_{*l}$ and for $\varPhi _{\theta }$ additionally the temperature flux $\overline{w'\theta '}$. The characteristic velocity $u_{*l}$ is included in both $\varPhi _{\theta }$ (or $\varPhi _q$) and $z/\varLambda$, potentially producing a spurious self-correlation between them. Self-correlation can produce erroneous confidence in scaling, so it should be examined before any attempt is made to apply local scaling (or MOST) to a dataset (Klipp and Mahrt 2004). The assessment of self-correlation has been investigated in the past (e.g., Hicks 1981; Andreas 2002; Klipp and Mahrt 2004; Nadeau et al. 2013a). Baas et al. (2006) found that self-correlation in the case of the non-dimensional gradient of mean wind speed ($\varPhi _m$) is significant, and can lead to misleading results. Nadeau et al. (2013a) used the method of Klipp and Mahrt (2004) for the dimensionless standard deviations of wind speed, temperature and humidity to find significant self-correlation of the horizontal velocity components for unstable and stable conditions.

**Fig. 7:**
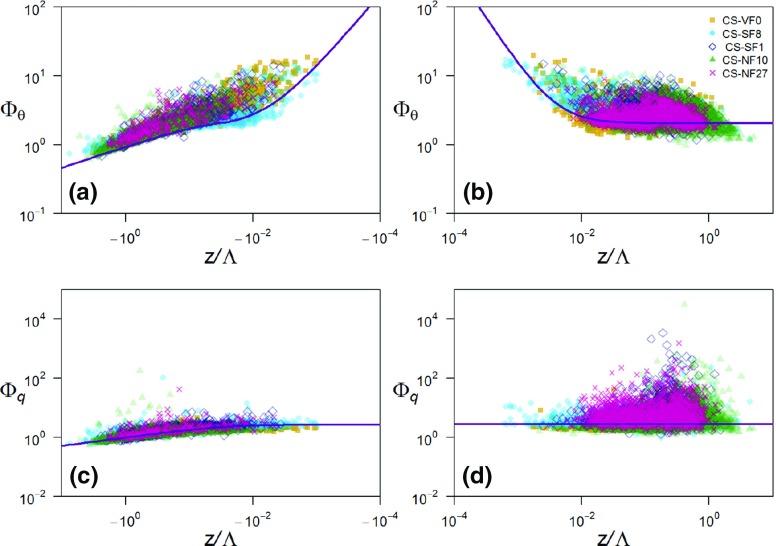
Non-dimensional temperature (**a**, **b**) and humidity (**c**, **d**) standard deviations as a function of local stability, for unstable (left) and stable stratification (right) for the i-Box sites. Reference curves are shown in purple

The existence of self-correlation in $\varPhi _{\theta }$ and $\varPhi _q$ as a function of stability is herein assessed for every i-Box site and for every measurement level. Using the Klipp and Mahrt (2004) method, we apply a robust linear regression between $\varPhi _{\theta }$ and $\varPhi _q$ and $z/\varLambda$. The variables $u_{*l}$, $\sigma _{\theta }$, $\sigma _q$, $\overline{w'\theta '}$ and $\overline{w'q'}$ are then initially randomized 1000 times, before calculating the mean random correlation coefficient $R_{rand}$ between $\varPhi _{\theta }$ and $z/\varLambda$ ($\varPhi _q$ and $z/\varLambda$), and comparing with the correlation coefficient of the original data series, $R_{data}$. If the absolute difference $|R_{data}^2-R_{rand}^2| \approx 0$, this implies that the randomized dataset exhibits a similar degree of correlation as the true variables, suggesting significant self-correlation. In contrast, if $|R_{data}^2-R_{rand}^2|>> 0$, then either $R_{data}>> R_{rand}$ (insignificant self-correlation) or $R_{rand}>>R_{data}$. The second case occurs when the original dataset is not well correlated itself (as in Fig. 7d), so that the randomized dataset has a higher correlation, implying insignificant self-correlation again. In other words, the obtained relationship is not predominately affected by self-correlation if the values of $R_{data}$ and $R_{rand}$ differ strongly.

As no study exists that provides a specific quantitative threshold for $|R_{data}^2-R_{rand}^2|$ to indicate self-correlation, we calculate the normalized difference $K = (R_{data}^2-R_{rand}^2)/R_{data}^2$, which indicates the fraction of the variance explained by physical processes. When $K \approx 0$, the dataset is suspected to be self-correlated. However, we avoid defining a certain threshold for *K*, because that would be arbitrary and dependent on the available datasets. In the case of $\varPhi _{\theta }$, the degree of self-correlation is checked separately in the near-neutral region and in the region with stronger stability, because of the different slopes of the similarity functions in unstable and stable stratification, given that we apply linear robust regression.

Table 4 summarizes the results for $\varPhi _{\theta }$, where $R_{data}$ values are in all cases much larger than $R_{rand}$ values in unstable stratification, and $K>> 0$, indicating that self-correlation is not dominating the $\varPhi _{\theta }$ relationship. On the unstable side of near-neutral stratification, *K* is in almost every case close to zero, indicating a considerable influence of self-correlation, but $R_{data} > R_{rand}$ in the majority of cases. It is noted that the value of *K* is positive for all unstable cases.

On the stable side ($z/\varLambda \ge 0.05$), the value of *K* is large in magnitude for all i-Box sites, but with $R_{rand} > R_{data}$, probably because of the very small slope of the best-fit curves in this region. The horizontal best-fit curves show that there is no actual dependence of $\varPhi _{\theta }$ on $z/\varLambda$, so any random dataset will have a larger correlation coefficient $R_{rand}$. The near-neutral (stable) region exhibits relatively small absolute values of *K*, albeit with the majority being somewhat larger than on the unstable side.

Overall, the results of Table 4 indicate that self-correlation is not the dominant factor influencing the $\varPhi _{\theta }(z/\varLambda )$ relationships for non-near-neutral stratification. In the near-neutral range (especially on the unstable side), the value of *K* is close to zero, indicating that the obtained functional relationships are largely influenced by self-correlation.

Concerning $\varPhi _q$, as mentioned before, there is no discrimination between stronger and near-neutral stabilities. From Table 5, it can be seen that *K* is larger for unstable than for stable stratification. On the unstable side, the values of *K* vary between $- 0.26$ and 0.55 among the measurement sites, suggesting weak self-correlation. The strongly negative values of *K* in the stable range are likely due to the large scatter of the data points and not to self-correlation. Hence, the above results indicate that, for the relationship between $\varPhi _q$ and $z/\varLambda$, self-correlation is not a dominant factor.

**Fig. 8:**
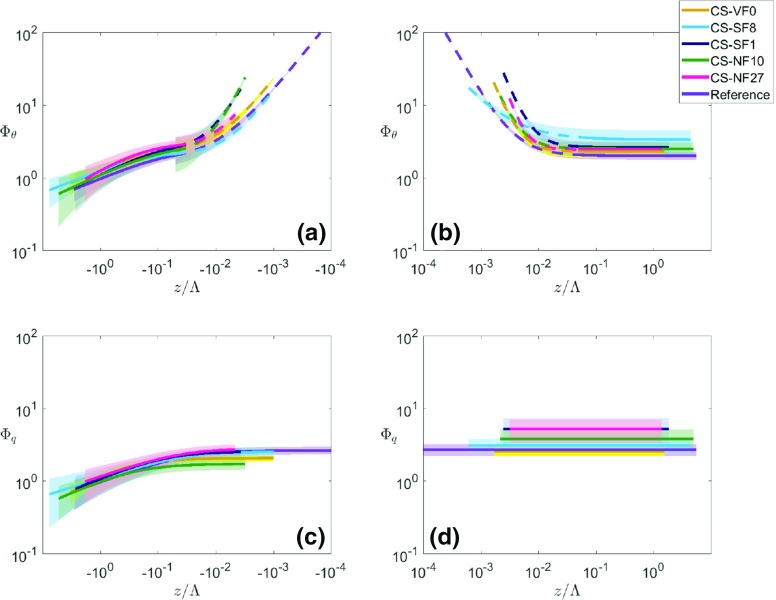
Best-fit curves of non-dimensional temperature (**a**, **b**) and humidity (**c**, **d**) standard deviations as a function of local stability for unstable (left) and stable stratification (right) for the i-Box sites. Dashed lines correspond to regions that are possibly affected by self-correlation. Shaded: areas between $+$ *MAD* and − *MAD* values of each i-Box dataset,the where the *MAD* variable represents is the median absolute deviation. Note that, due to the logarithmic representation, the width of the shaded area changes along the *x*-axis at the threshold between near-neutral and stronger instability for $\varPhi _{\theta }$

Although $\varPhi _{\theta }$ was found to be seriously affected by self-correlation in the near-neutral regions of stable and unstable stratifications, the corresponding data are not excluded from the present analysis because it is nevertheless considered useful to study the impact of terrain complexity under these conditions. In particular, when inspecting the degree of self-correlation at the reference site (Tables 4, 5), it becomes clear that the degree of self-correlation does not seem to be primarily a question of terrain complexity. However, since the results from stability ranges potentially dominated by self-correlation should be considered with necessary caution, we graphically distinguish those results from those less affected (see Fig. 8).

The present assessment also reveals that the self-correlation test of Klipp and Mahrt (2004) yields results that are difficult to interpret when applied to a non-linear functional, or at least near-linear. Generalizing, however, the self-correlation test to non-linear relations is beyond the scope of the present study.

**Table 4:** Assessment of self-correlation between the non-dimensional temperature standard deviation and local stability for all i-Box sites, for unstable and stable conditions (using robust linear-regression coefficients); *N* is the number of data points, $R_{data} = \sqrt{R_{data}^2}$ and $R_{rand} = \sqrt{R_{rand}^2}$ are the linear correlation coefficients of the real data and the randomized data, respectively, and $K = (R_{data}^2-R_{rand}^2)/R_{data}^2$ is the fraction of the variance explained by physical processes

Unstable		$z/\varLambda \le -0.05$				$-0.05 < z/\varLambda \le 0$			
Site	Level	*N*	$R_{data}$	$R_{rand}$	*K*	*N*	$R_{data}$	$R_{rand}$	*K*
	1st	1969	0.79	0.46	0.66	657	0.69	0.62	0.2
CS-VF0	2nd	2341	0.77	0.54	0.51	303	0.59	0.54	0.16
	3d	2385	0.7	0.58	0.31	252	0.66	0.57	0.27
CS-SF8	1st	940	0.86	0.5	0.66	390	0.89	0.66	0.44
	2nd	992	0.85	0.57	0.54	228	0.62	0.62	0.02
CS-SF1	1st	684	0.78	0.6	0.42	94	0.52	0.48	0.15
CS-NF10	1st	1001	0.78	0.59	0.42	91	0.62	0.6	0.035
CS-NF27	1st	306	0.68	0.54	0.37	58	0.5	0.49	0.05
Reference (Cabauw)	1st	1169	0.88	0.44	0.75	1750	0.92	0.89	0.055

Stable		$0\le z/\varLambda < 0.05$				$z/\varLambda \ge 0.05$			
Site	Level	*N*	$R_{data}$	$R_{rand}$	*K*	*N*	$R_{data}$	$R_{rand}$	*K*
	1st	824	0.67	0.59	0.21	1299	0.34	0.46	− 0.84
CS-VF0	2nd	348	0.45	0.54	$-0.48$	1760	0.28	0.49	− 2.01
	3d	304	0.62	0.54	0.23	1811	0.3	0.51	− 2.01
CS-SF8	1st	337	0.39	0.57	$-1.11$	207	0.36	0.49	− 0.8
	2nd	264	0.68	0.57	0.29	390	0.2	0.49	− 4.78
CS-SF1	1st	149	0.71	0.51	0.49	1119	0.26	0.5	− 2.7
CS-NF10	1st	202	0.69	0.52	0.44	2117	0.29	0.57	− 2.9
CS-NF27	1st	567	0.5	0.57	$-0.28$	1599	0.25	0.51	− 3.28
Reference (Cabauw)	1st	2693	0.89	0.76	0.28	2510	0.3	0.47	− 1.48

**Table 5:** Assessment of self-correlation between the non-dimensional humidity standard deviation and local stability for all i-Box sites, for unstable and stable conditions (robust linear-regression coefficients)

Site	Level	Unstable				Stable			
		*N*	$R_{data}$	$R_{rand}$	*K*	*N*	$R_{data}$	$R_{rand}$	*K*
CS-VF0	1st	2626	0.79	0.53	0.55	2123	0.6	0.84	− 0.94
	3d	2637	0.73	0.64	0.23	2115	0.69	0.76	− 0.2
CS-SF8	2nd	1220	0.75	0.67	0.21	654	0.81	0.66	0.32
CS-SF1	1st	778	0.71	0.52	0.47	1268	0.85	0.87	− 0.048
CS-NF10	1st	1092	0.73	0.82	− 0.26	2319	0.56	0.69	− 0.51
CS-NF27	1st	364	0.66	0.64	0.078	2166	0.82	0.91	− 0.24
Reference (Cabauw)	1st	2919	0.8	0.79	0.01	5203	0.93	0.56	0.64

Variables as in Table 4

## Results

### Scaled Temperature Standard Deviation

The similarity functions for the temperature standard deviation are presented and discussed here. In Table 6 of the Appendix, the non-linear best-fit functions between $\varPhi _{\theta }$ and $z/\varLambda$ are shown for every i-Box site for unstable ($z/\varLambda \le -0.05$), near-neutral unstable ($- 0.05 < z/\varLambda \le 0$) and stable ($z/\varLambda \ge 0$) conditions. The number of available data points for every stability region is also listed. The derived best-fit similarity functions are only from the first level of every i-Box site. It should be noted, however, that the differences in the obtained best-fit relations between different heights where available (the CS-VFO and CS-SF8 sites) are in all aspects similar to those between sites (not shown). Tables 4 and  6 show that the number of available data points varies between the i-Box sites because of many missing or excluded data due to instrument malfunction during the operating period or low data quality. As expected, the small number of available data points increases the uncertainty in the parametrizations, especially in the near-neutral ranges, which also gives an increased possibility of self-correlation. However, these ranges are not excluded from the analysis as the fitted equations in the near-neutral region indicate the possible impact of terrain inhomogeneity.

Table 6 lists the observed range of $z/\varLambda$ for every site, with $|z/\varLambda |$ always much smaller than 10. In contrast, $|z/\varLambda |$ reaches very small values in the near-neutral range (within the measurement uncertainty), with the smallest value ($- 9.7\times 10^{-4}$) occurring at the south-facing CS-SF8 site.

For the best-fit analysis, all the coefficients in the general formulation $a_{\theta }$, $b_{\theta }$, $c_{\theta }$, $d_{\theta }$ and $e_{\theta }$ are fitted parameters based on Eq. 5 for $i = \theta$,

6 $$\begin{aligned} \varPhi _{\theta } = \left\{ \begin{array}{ll} a_{{\theta }u}(b_{{\theta }u}-z/\varLambda )^{d_{{\theta }u}} &{}\quad \text{ for } \quad z/\varLambda \le -0.05 \\ a_{{\theta }n}(-z/\varLambda )^{d_{{\theta }n}}+e_{{\theta }n} &{}\quad \text{ for } \quad -0.05 < z/\varLambda \le 0 \\ a_{{\theta }s}z/\varLambda ^{d_{{\theta }s}}+e_{{\theta }s} &{}\quad \text{ for } \quad z/\varLambda \ge 0. \\ \end{array} \right. \end{aligned}$$

The coefficients $c_{{\theta }u} = c_{{\theta }n} = c_{{\theta }s}$ and $e_{{\theta }u} = b_{{\theta }n} = b_{{\theta }s}$ (see Eq. 5) are set to $- 1$ and to zero, respectively, as these values have also been used in other studies (e.g., Tillman 1972; Tampieri et al. 2009; Pahlow et al. 2001). The subscripts *u*, *n*, *s* refer to unstable, near-neutral and stable ranges, respectively. The best-fit coefficients for every i-Box dataset are calculated by applying a non-linear robust fit with a bi-squared weighting, as in the case of the reference curves.

The statistical differences between individual i-Box datasets, and also between the i-Box and the reference datasets, are examined by applying the Kolmogorov–Smirnov test for every stability range separately. The purpose of this nonparametric test is to examine whether differences in the cumulative distributions of two datasets are statistically significant at the significance level of $5\%$. For this purpose, the same stability range and the same number of data points are considered for the compared datasets. To compare two different-sized datasets, the same number of data points is obtained by randomizing the dataset with the larger number of data points using the Bootstrap method, and randomly choosing the same number of data points as in the second dataset. The above test shows statistically significant differences between all i-Box datasets and the reference for unstable, stable and near-neutral (unstable) stratification. However, between individual i-Box datasets, differences in distributions are not statistically significant in some cases, as is the case between the sites CS-NF10, CS-NF27 and CS-SF1 in the near-neutral (unstable) range, as well as between the CS-SF1 and CS-NF27 sites in the unstable range. For stable stratification, differences between all the i-Box datasets are found to be statistically significant. The few exceptions (four out of 48 pairs of datasets) suggest that the differences between the $\varPhi _{\theta }$ distributions of the different sites are generally statistically significant.

In Fig. 7, the five i-Box datasets are plotted together with the reference curves, with the panels (a) and (b) of Fig. 7 showing the differences in $\varPhi _{\theta }(z/\varLambda$) between the sites for unstable and stable stratifications, respectively. It can clearly be seen that, on average, $\varPhi _{\theta }$ is larger than the reference for all the i-Box sites outside the near-neutral range for unstable and stable stratifications, as already noted by Rotach et al. (2017). Specifically, in the unstable region, the best-fit curve of the steepest mountain-slope site (the CS-NF27 site) exhibits the largest $\varPhi _{\theta }$ values (Fig. 8a). However, the magnitudes of the best-fit curves for the i-Box sites do not seem to be proportional to the mountain slope. In the near-neutral (unstable) region, the curve slopes for the i-Box datasets differ from the reference. This result is better depicted in Fig. 8a where only the slope of the best-fit curve for the CS-SF8 site is smaller than the reference, while all the other curve slopes are larger than the reference, with the CS-NF10 and CS-SF1 sites having the largest curve slopes for unstable stratification. It should be noted that the CS-SF1 site is highly inhomogeneous, as it is surrounded by grass fields to the east, a house to the north, and a corn field on an escarpment to the west.

On the stable side in the near-neutral region (Fig. 8b), the CS-NF10 and CS-SF1 sites have the largest deviation from the reference, although there is no clear dependence of the slope of the curves on the terrain slope. For the region with stable stratification, the best-fit curves for the CS-VF0 and CS-NF27 sites are closest to the reference curve (Fig. 8b, Table 6). In the Appendix (Fig. 13), plots of $\varPhi _{\theta }$($z/\varLambda$) and $\varPhi _q$($z/\varLambda$) are shown for every site separately. On the strongly stable side, it can be seen that the best-fit i-Box and reference curves become horizontal, indicating a small dependence of $\varPhi _{\theta }$ on $z/\varLambda$ (Fig. 8b). This is expected, as for large values of $z/\varLambda$, the weak turbulence inhibits any exchange with the surface and, therefore, the scaled variable $\varPhi _{\theta }$ becomes independent of $z/\varLambda$, corresponding to *z*-less scaling (Nieuwstadt 1984).

According to the best-fit equations for the i-Box sites in the near-neutral (unstable) range, the exponent $d_{{\theta }n}$ is smallest for the valley-floor site (CS-VF0) and largest for the steep-sloped site (CS-NF10), although the coefficient does not increase proportionally to the mountain slope (Table 6). For stable stratification, the exponent $d_{{\theta }s}$ is smallest for the site CS-SF8 and largest for the site with the steepest slope (CS-NF27). The largest differences in the curves’ slopes between the i-Box sites are detected in the near-neutral range of both unstable and stable stratifications (Fig. 8a, b, Table 6). With stronger stability (for both unstable and stable stratifications), the best-fit curves of the i-Box sites are relatively close to each other.

To examine whether the scatter around the best-fit curves is so large that the curves are not significantly different, we test whether a similarity curve from one site can be used for sites with different surface characteristics. For this purpose, the scatter of the data around the i-Box best-fit and reference curves is illustrated in Fig. 8, with shaded areas depicting the scatter of each dataset based on the median absolute deviation (*MAD*) of the data. The value of *MAD* is calculated for every stability range (unstable, near-neutral and stable) and for every site separately. These shaded areas represent the confidence intervals for each best-fit curve. It should be noted that the confidence intervals differ in width along each best-fit curve, because of the logarithmic representation. In the strongly unstable range of Fig. 8a ($z/\varLambda < -1$), the confidence intervals overlap, because of the large scatter of each dataset. However, for $- 1 \le z/\varLambda < -0.05$, it can be seen that the confidence interval for the CS-NF27 site does not overlap with that for the reference site. In the near-neutral (unstable) range, the confidence intervals are not overlapping, except for site CS-NF10 with site CS-SF1. Therefore, despite the uncertainty in the datasets, the parametrizations are unique for every dataset in the near-neutral (unstable) range. For strongly stable stratification, the confidence intervals overlap, except for site CS-SF8, whose best-fit curve is much higher than all the others (Fig. 8b). However, in the near-neutral (stable) range, these intervals are separated from each other, emphasizing the uniqueness of each best-fit curve, which confirms our initial hypothesis that the universal MOST equations are not suitable for complex terrain, as the similarity functions we found are strongly site dependent.

As mentioned before, standard deviations of the non-dimensional temperature for the i-Box sites have larger magnitudes than those from the reference site, which is especially true for the unstable range. Figure 1 shows that this is also the case for all the HIF and complex-terrain cases from the literature review for stable stratification, but only for a limited number of cases in the unstable range. Comparing the $\varPhi _{\theta }$ best-fit equations of Cabauw and the i-Box sites (Table 6), it can be seen that, for unstable stratification, the coefficients $a_{{\theta }u}$, which determine the magnitudes of the curves, are larger for the i-Box sites than for the reference site. Similarly, in the stable region, the coefficients $e_{{\theta }s}$, which determine the curves’ magnitudes—because $a_{{\theta }s}$ is very small for all sites—are much larger for all i-Box sites than for the reference site. In Fig. 8, it is shown that the differences between the i-Box and the reference best-fit curves are substantial. The question arises as to what the possible reasons for this enhanced temperature variability may be compared with ‘ideal’ sites, i.e., larger $\varPhi _{\theta }$ values at sites over complex terrain.

**Fig. 9:**
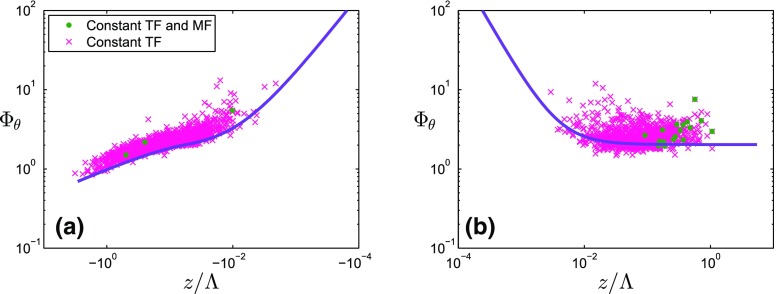
Non-dimensional temperature standard deviation as a function of local stability at the first level of the CS-VF0 site for unstable (left) and stable stratifications (right) when the fluxes are constant with height (*TF* temperature fluxes, *MF* momentum fluxes). Reference curves are shown in purple

**Fig. 10:**
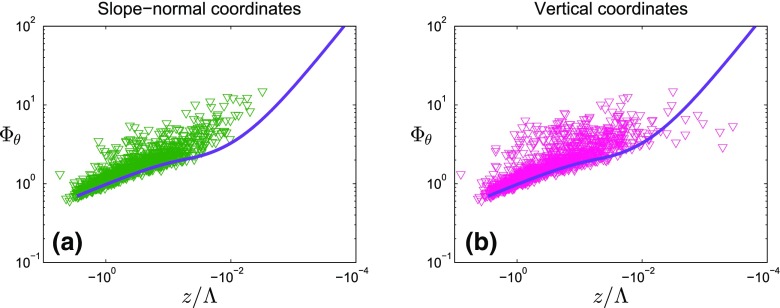
Non-dimensional temperature standard deviation as a function of local stability for the CS-NF10 site, with slope-normal coordinates (**a**) and vertical coordinates (**b**) for unstable stratification. Reference curves are shown in purple

We have identified four possible reasons for this:

- Post-processing: as i-Box and reference datasets were both analyzed with exactly the same post-processing method by the EdiRe software, with $\varPhi _{\theta }$ only found to be much larger than in the HHF and HIF curves (e.g., Kaimal and Finnigan 1994; Tillman 1972) for the i-Box dataset, and not for the reference, this excludes the post-processing from being the reason.
- The zero-plane displacement is determined in a relatively crude manner (see Sect. 3.1). However, when using extreme values for the zero-plane displacement (e.g., $d = 0$, or double the estimated values), no significant differences from the original values shown in Fig. 8 and Table 6 are observed (not shown), which suggests that the estimation of the zero-plane displacement is not the reason for the large values of $\varPhi _{\theta }$.
- Non-constant fluxes: Nadeau et al. (2013a) used the non-constancy of the turbulent fluxes as one of the arguments as to why the MOST approach may not be applicable in complex topography (but rather local scaling). While we certainly agree with this argument, we investigated whether periods of ‘non-constant fluxes’ exhibit particularly large $\varPhi _{\theta }$ values. Figure 9 shows that the data points with constant fluxes are not closer to the reference curves than data for non-constant fluxes, for both unstable and stable stratifications at the CS-VF0 site. This result confirms that the difference from the reference curves for temperature, which is observed at each i-Box site, is not due to the existence of non-constant fluxes.
- Coordinate system (i.e., the frame of reference for the projection of the temperature and momentum fluxes): while it is customary to use a terrain-following coordinate system over sloped surfaces, so that the temperature and momentum fluxes are normal to the surface, this introduces some difficulty because even if the local (perturbation) isentropes are parallel to the slope, the dominant direction of heat fluxes at some distance away from the surface is vertical (Stiperski and Rotach 2016; Oldroyd et al. 2016a, b; Lobocki 2017). We have, therefore, tested the hypothesis that the vertical (rather than the normal) heat fluxes constitute the appropriate scaling variable by comparing $\varPhi _{\theta }$ calculated with slope-normal and vertical temperature fluxes for the four i-Box sites with sloping terrain (Figs. 10, 14 in the Appendix). The fluxes are converted from slope-normal to vertical coordinates, following the method of Oldroyd et al. (2016a). Figure 10 shows that this coordinate transformation does not affect the magnitude of $\varPhi _{\theta }$ on the unstable side, but does affect the curve’s slope in the near-neutral (unstable) region. In the Appendix (Fig. 14), it can be seen that both sites with steeply sloping terrain (the CS-NF10 and CS-NF27 sites) exhibit this decrease of the curve slope in the near-neutral (unstable) range when using vertical temperature fluxes, although there are not enough data points to substantiate this statement. For the sites CS-SF1 and CS-SF8, this phenomenon is not observed systematically. Despite this decrease in the curves’ slopes, it is clear that the large majority of i-Box data points in the unstable region do not change after axis transformation.
  According to Oldroyd et al. (2016a), the change from slope-normal to vertical coordinates may lead to a significant change in $z/\varLambda$ under stable conditions, and even a change in sign (while the thermal stratification remains stable). Indeed, when moving from slope-normal to vertical coordinates according to the approach of Oldroyd et al. (2016a), a change of sign is detected in about 100 cases at the CS-NF10 site, and for a few at the other sites. More importantly, on the stable side, the move from slope-normal to vertical coordinates changes the distribution of $\varPhi _{\theta }$ data points (not shown) in the sense that the scatter of the datasets increases, especially at the strongly-sloped sites (the CS-NF10 and CS-NF27 sites). This leads to the separation of the datasets into two main clusters for the sites CS-NF10 and CS-NF27, likely because of two different flow types (i.e., katabatic vs. dynamically-modified flows under stable conditions). It does not, however, alter the fact that $\varPhi _{\theta }$ is systematically larger than predicted from the reference curve.

To conclude, none of the four reasons discussed can be responsible for the large values detected in the plots of (especially) the unstable $\varPhi _{\theta }$($z/\varLambda$) values (Fig. 7). Therefore, we conclude that the inhomogeneity of the study area and the complexity of the terrain are likely the reasons for this difference.

### Scaled Humidity Standard Deviation

In Table 6 (in the Appendix), the best-fit coefficients for the five i-Box sites are shown, together with the available number of data points for every region. The unstable side is not divided into unstable and near-neutral (unstable) regions, because there is no near-neutral variability, as in the case of $\varPhi _{\theta }$. For stable stratification, only one coefficient is fitted to the data. Due to the large scatter in the stable range (Fig. 7d), the best-fit curve appears to be a horizontal line, indicating no significant dependence of $\varPhi _q$ on $z/\varLambda$. As for $\varPhi _{\theta }$, the coefficients $a_q$, $b_q$ and $d_q$ in the similarity functions were fitted to the data for unstable stratification, so that differences in the slope and the magnitude of the curves can be shown. To compare the best-fit equations of $\varPhi _{\theta }$ and $\varPhi _q$ (Sect. 6.3), we consider the same general formulation for $\varPhi _q(z/\varLambda )$ as for $\varPhi _{\theta }(z/\varLambda )$ in the unstable region,

7 $$\begin{aligned} \varPhi _q = \left\{ \begin{array}{ll} a_{qu}(b_{qu}-z/\varLambda )^{d_{qu}} &{} \quad \text{ for } \quad z/\varLambda \le 0 \\ e_{qs} &{} \quad \text{ for } \quad z/\varLambda \ge 0 \\ \end{array} \right\} , \end{aligned}$$

where the subscripts *u* and *s* denote unstable and stable conditions, respectively. In Fig. 7c, d, $\varPhi _q$ as a function of the stability is shown, and for a better distinction between the sites, Fig. 13 in the Appendix depicts each i-Box dataset separately. On the unstable side, the sites with steep-terrain slopes (the CS-NF10 and CS-NF27 sites) present high scatter, whereas the CS-VF0 dataset has the smallest scatter around the reference curve (Fig. 7c). In contrast to temperature, the $\varPhi _q$ curves do not generally exhibit a larger magnitude than the reference curve, illustrating why the best-fit coefficients $a_{qu}$ of the i-Box curves are similar to the coefficient for the reference best-fit (cf. Table 6). In the near-neutral region of the unstable range, data show no increasing scatter for all the i-Box sites (Fig. 7c).

Figure 8c shows that the best-fit curve of the CS-NF27 site is always higher in magnitude than the reference on the unstable side, whereas the other i-Box best-fit curves are higher in the strongly unstable range, but smaller in magnitude than the curves reported in the literature for $z/\varLambda > -0.1$. In accordance with the literature review (e.g., Andreas et al. 1998; Liu et al. 1998), the chosen formulation for the best-fit function for stable stratification is a constant, which is also suggested by the scatter of the present data. In Fig. 8d, the CS-NF27 and CS-SF1 site curves are the highest in magnitude ($\varPhi _q = 5.28$ and $\varPhi _q = 5.25$, respectively), whereas for the other sites, the value of $\varPhi _q$ decreases, following the decrease of the terrain slope. Andreas et al. (1998) found $\varPhi _q = 4.1$ as the best-fit for stable stratification in the case of metre-scale heterogeneous terrain, whereas Liu et al. (1998) found $\varPhi _q = 2.4$ in the case of HHF terrain. For the i-Box sites, best-fit curves of $\varPhi _q$ are found to vary between 2.58 and 5.28 (Table 6). It should be noted that, in all cases, the best-fit curve of the CS-VF0 site is closer to the reference curve than those of the other i-Box sites. All of the above suggests that the magnitude of $\varPhi _q$ for stable stratification is affected by the terrain slope or the heterogeneity of the terrain, without, however, any direct relationship between them.

The application of the Kolmogorov–Smirnov test shows that almost all differences in dataset distributions are statistically significant. For unstable stratification, the differences in data distribution are not statistically different between the sites CS-SF1 and CS-SF8, as well as between sites CS-NF27 and CS-SF8 (note that the statistically similar site pairs are not the same as those for temperature). These few exceptions again suggest that, overall, the differences between the $\varPhi _q$ distributions of the different sites are statistically significant.

The shaded areas around the best-fit curves in Fig. 8c, d represent the *MAD* values of the datasets and, therefore, the confidence intervals of the best-fit curves. In Fig. 8c, it is noted that almost all the confidence intervals overlap for strong instability. In contrast, the intervals for sites CS-VF0 and CS-NF10 start to diverge from the rest for $z/\varLambda > -0.1$. For stable stratification in Fig. 8d, the high scatter of all datasets causes all the confidence intervals to overlap. It should be noted that the confidence interval for the CS-VF0 site is almost the same as the reference site, as their best-fit curves are very similar, and both exhibit little scatter in the data.

### Comparison Between Temperature and Humidity Similarity Functions

Many studies have suggested using the best-fit function of $\varPhi _{\theta }$ for $\varPhi _q$, because the characteristics of humidity and temperature fluctuations are considered to be similar (e.g., Ramana et al. 2004). Here we investigate how useful this is by comparing the best-fit similarity curves of temperature and humidity for unstable and stable stratifications ($|z/\varLambda | \ge 0.05$). The bootstrapping method, combined with the Student’s *t*-test, is followed for all the cases to determine whether the differences between the two similarity functions are statistically significant. It should be noted that the temperature and humidity curves are not compared in the near-neutral regions, since the slope of the curves and the form of the functions are different there anyway.

**Fig. 11:**
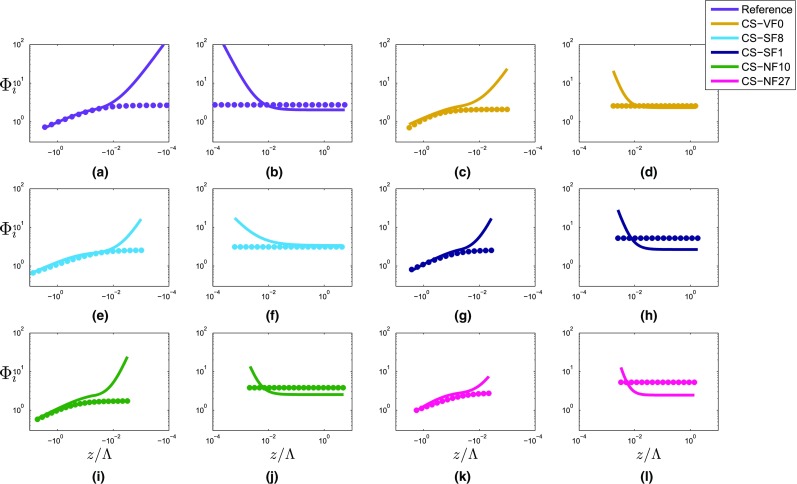
Comparison between best-fit curves of non-dimensional temperature (solid line) and humidity (symbols) standard deviations as a function of local stability for all sites

The differences between $\varPhi _{\theta }$ and $\varPhi _q$ for unstable and stable stratification are shown in Fig. 11. For unstable stratification, the best-fit curves for $\varPhi _{\theta }$ and $\varPhi _q$ are very similar for the sites with a small terrain slope (the reference, CS-VF0, CS-SF8 and CS-SF1 sites). In contrast, the slopes of the two curves differ noticeably for the CS-NF10 site, as well as in magnitude for the CS-NF27 site. In the near-neutral (unstable) range, the differences in slopes of the curves are large as expected (see Fig. 8a, c, e, g, i, k). For stable stratification, the $\varPhi _q$ curves are much higher in magnitude than $\varPhi _{\theta }$ for most of the sites (see Fig. 11b, d, f, h, j, l), exceptions being the CS-VF0 site, which gives almost identical curves, and the CS-SF8 site where the $\varPhi _q$ curve is lower in magnitude. The largest differences in the curves’ magnitudes are noted for the CS-SF1 and CS-NF27 sites. For the near-neutral (stable) regions, the slopes for $\varPhi _q$ are zero, whereas for $\varPhi _{\theta }$, they are larger than zero; therefore, the curves in this region are not compared.

By applying the bootstrapping method with the Student’s *t*-test, the best-fit coefficients $a_{{\theta }u}$, $b_{{\theta }u}$, $d_{{\theta }u}$ of $\varPhi _{\theta }$ are compared with the coefficients $a_{qu}$, $b_{qu}$, $d_{qu}$ of $\varPhi _{qu}$, respectively, for unstable stratification ($z/\varLambda \le -0.05$). For stable stratification ($z/\varLambda \ge 0.05$), the coefficients $e_{{\theta }s}$ are compared with the coefficients $e_{qs}$ for all the sites. The above analysis shows that most of the best-fit coefficients differ statistically significantly, the exception being the difference between $e_{{\theta }s}$ and $e_{qs}$ values for the reference site, indicating that, overall, the curves $\varPhi _{\theta }$($z/\varLambda$) and $\varPhi _q$($z/\varLambda$) are different for both stable and unstable stratifications when considering sites in complex terrain.

As a second step, the confidence intervals of each best-fit curve were calculated (not shown), and represent the areas between the 10th and the 90th quantile of the set of all possible best-fit curves derived from randomizing the datasets 1000 times. Even with these large quantiles, the confidence intervals of the best-fit curves do not overlap, which supports the conclusion that differences in the best-fit curves of $\varPhi _{\theta }$ and $\varPhi _q$ as a function of $z/\varLambda$ are statistically significant. As a result, the use of the $\varPhi _{\theta }$ similarity functions for $\varPhi _q$ is not recommended, especially in complex terrain.

## Summary and Conclusions

Our objective was to examine the applicability of local scaling to flux-variance relationships of temperature and humidity in the complex terrain of an Alpine valley. For this we used the i-Box dataset, consisting of data from five different sites of different slope angle, orientation and roughness, located in one of the major valleys in the Alps. As a reference for horizontally (weakly) inhomogeneous and flat terrain, an 11-month dataset from the Cabauw site in the Netherlands was used.

For the scaled standard deviation of temperature $\varPhi _{\theta }$, the reference formulation is based on Eq. 5 for strong stability ($|z/\varLambda | \ge 0.05$) and the suggestion of Tampieri et al. (2009) for the near-neutral range to use a curve of slope $- 1$. Data from the Cabauw study area agree with the ‘classical’ formulations from the literature for horizontally homogeneous and flat terrain in magnitude and slope in the range of their respective applicability, which is also true for the non-dimensional standard deviation of humidity ($\varPhi _q$).

The classical Monin–Obukhov equations for $\varPhi _{\theta }$ are not applicable to the reference dataset, which do not account for the slope of the reference-data curve in the near-neutral (unstable) region. For the data outside the near-neutral range ($|z/\varLambda | \ge 0.05$), the reference best-fit curves are found to be similar to results from previous studies for HHF terrain, and, therefore, were used as a reference here. Although the same quality control and corrections were applied to both the reference dataset and to the i-Box data, the reference data fitted perfectly to the Tillman (1972) curve on the unstable side, but the i-Box data points lie mostly above this curve (Fig. 7).

The i-Box sites are characterized by turbulent fluxes that usually vary with height, considering the strong requirement that all turbulent fluxes have to be simultaneously constant with height. Specifically, the value of $\overline{w'\theta '}$ is only found to be approximately constant with height about 50$\%$ of the time, while momentum fluxes are usually height dependent for the study period. Although only two sites were examined for the constant-flux hypothesis (one at the valley floor and one with a weak slope), it can be safely assumed that, if the constant-flux hypothesis fails at the valley floor, which approaches HHF terrain to some degree, it will likely also fail for the mountain slopes. Since constant turbulent fluxes with height are an assumption of Monin–Obukhov similarity, the basic research question was the applicability of local scaling in highly complex terrain.

The self-correlation test for $\varPhi _{\theta }$ and $\varPhi _q$ as a function of $z/\varLambda$ following Klipp and Mahrt (2004) shows that data are not self-correlated outside the near-neutral range. The near-neutral data for both stable and unstable stratification, however, showed self-correlation (Tables 4, 5). Although the near-neutral regions probably ‘suffer’ from self-correlation, data from these regions were not excluded from the analysis in order to investigate their dependence on the inhomogeneity of the study area. It should be mentioned though that the results from the near-neutral regions should be treated with caution, because either the currently available methods for assessing self-correlation (e.g., Klipp and Mahrt 2004) need improvement, or a completely new method is needed to represent data with non-linear relationships more adequately. Also, conditions for a threshold for $R_{data}^2-R_{rand}^2$ should be defined to allow a more quantitative determination of self-correlation.

The analysis of the $\varPhi _{\theta }$ similarity functions to i-Box data indicates that the best-fit similarity functions for every i-Box site are different, and these differences are statistically significant (Fig. 8, Table 6). While similarity curves differ in terms of both slope and magnitude of the curves, some similarities—such as a large slope of the curve in the near-neutral region—are found between the sites with the steepest terrain slopes (the CS-NF10 and CS-NF27 sites). Furthermore, the best-fit curve of the valley-floor site (the CS-VF0 site) is found to be more similar to the reference curve than the other sites for stable stratification, but still (in a statistical sense) significantly different (Fig. 8b, Table 6).

Similarity functions for $\varPhi _{\theta }$ were found to be significantly larger in magnitude than the reference at all sites, and for stabilities outside the near-neutral (unstable) range. Four possible reasons for this were investigated: the post-processing method, the determination of the zero-plane displacement, the non-constancy of the fluxes with height, and the slope-following coordinate system. As shown in the results, significantly larger $\varPhi _{\theta }$ values than the reference are caused neither by the post-processing method, nor by the non-constancy of the fluxes. It is further demonstrated that the large values are not due to the crude method used to determine the zero-plane displacement in $z/\varLambda$, or the chosen coordinate system. Having rejected these potential reasons, the most probable reason for this difference is the complexity of the terrain. It should be noted that all i-Box sites exhibit differences in the magnitude of the curve, even the valley-floor site, and so cannot be the result of the local terrain slope of the sites, but possibly the general terrain inhomogeneity of the study area.

In contrast to $\varPhi _{\theta }$, similarity functions for the scaled standard deviation of humidity $\varPhi _q$ as a function of stability for unstable conditions show no scatter of the data points in the near-neutral region in agreement with the literature review. However, the magnitude of the best-fit curves are affected by the mountain slope of each i-Box site (Fig. 8, Table 6). The magnitude of the best-fit curve of the i-Box site with the maximum mountain slope (the CS-NF27 site) is significantly higher than the curves for the other sites in the unstable range, and exhibits much larger scatter in the stable regimes. However, this difference, which is influenced by the mountain slope, is not evident in the slope of the curves in contrast to $\varPhi _{\theta }$. It should be noted that, although there is a remarkable difference in the magnitude of $\varPhi _q$ between the i-Box sites with a large mountain slope, and those with zero or a small slope, the similarity between the curves for the site with the largest local slope and the one with a minor slope suggests that this difference is not directly related to the terrain slope (or other site characteristics).

As a final step, the comparison between the best-fit curves of $\varPhi _{\theta }$ and $\varPhi _q$ was conducted for all sites for non near-neutral stratification (Fig. 11), illustrating that differences in the two types of curves are generally statistically significant for all cases, except for the reference curves, with differences in curve magnitude more profound for stable stratification. Additionally, as the differences are affected by the mountain slope of the site, it is not recommended to use $\varPhi _{\theta }$ to describe the humidity fluctuations as a function of $z/\varLambda$, especially in non-homogeneous terrain.

The failure of one of the basic assumptions of MOST concerning the independence of fluxes with height means that the application of local scaling is recommended. Therefore, results show that local scaling has some potential even in highly complex terrain, but with the disadvantage that the coefficients in the $\varPhi _i$ functions are site-specific. Consequently, as coefficients cannot be transferred to another study area (or even another site within the same area), the local characteristics of the similarity functions first need to be established before application of flux-variance similarity.

**Table 6:** List of best-fit similarity relations for the non-dimensional temperature $\varPhi _{\theta }$ and humidity $\varPhi _q$ standard deviations, for every i-Box site, for unstable, near-neutral (unstable) and stable stratifications

$$\varPhi _{\theta }$$								
i-Box site	*N*	$a_{\theta }$	$b_{\theta }$	$c_{\theta }$	$d_{\theta }$	$e_{\theta }$	Stability	Stability range
CS-VF0	1969	1.29	0.081	− 1	− 0.33	0	Unstable	$-3.3\le z/\varLambda \le -0.05$
	657	0.011	0	− 1	− 1.1	2.24	Near-neutral (unstable)	$-0.05< z/\varLambda \le -0.001$
	2123	$2.8\times 10^{-5}$	0	1	− 2.1	2.32	Stable	$1.7\times 10^{-3}\le z/\varLambda \le$ 1.53
CS-SF8	940	1.32	0.17	− 1	− 0.32	0	Unstable	$-7.59\le z/\varLambda \le -0.05$
	390	$3.6\times 10^{-3}$	0	− 1	− 1.2	2.025	Near-neutral (unstable)	$-0.05< z/\varLambda \le -9.7\times 10^{-4}$
	544	0.018	0	1	− 0.9	3.39	Stable	$6.1\times 10^{-4} \le z/\varLambda \le$ 4.38
CS-SF1	684	1.14	0.069	− 1	− 0.4	0	Unstable	$-2.71\le z/\varLambda \le -0.05$
	94	$3\times 10^{-3}$	0	− 1	− 1.5	2.35	Near-neutral (unstable)	$-0.05< z/\varLambda \le -3.7\times 10^{-3}$
	1268	$1.05\times 10^{-4}$	0	1	− 2.07	2.67	Stable	$2.5\times 10^{-3}\le z/\varLambda \le$ 1.84
CS-NF10	1001	1.13	0.081	− 1	− 0.37	0	Unstable	$-5.28\le z/\varLambda \le -0.05$
	91	$1.2\times 10^{-3}$	0	− 1	− 1.7	2.19	Near-neutral (unstable)	$-0.05< z/\varLambda \le -3\times 10^{-3}$
	2319	$6.8\times 10^{-5}$	0	1	− 1.96	2.54	Stable	$2.2\times 10^{-3} \le z/\varLambda \le$ 4.98
CS-NF27	306	1.38	0.2	− 1	− 0.5	0	Unstable	$-1.82\le z/\varLambda \le -0.05$
	58	$4.5\times 10^{-3}$	0	− 1	− 1.3	2.57	Near-neutral (unstable)	$-0.05< z/\varLambda \le -4.6\times 10^{-3}$
	2166	$3.37\times 10^{-6}$	0	1	− 2.6	2.46	Stable	$3.2\times 10^{-3} \le z/\varLambda \le$ 1.39
Reference (Cabauw)	1169	0.99	$6.3\times 10^{-2}$	− 1	v1/3	0	Unstable	$-2.87\le z/\varLambda \le -0.05$
	1750	$1.5\times 10^{-2}$	0	− 1	− 1	1.76	Near-neutral (unstable)	$-0.05< z/\varLambda \le -7\times 10^{-5}$
	5203	$8.7\times 10^{-4}$	0	1	− 1.4	2.03	Stable	$1.4\times 10^{-5} \le z/\varLambda \le$ 5.5

$$\varPhi _q$$								
i-Box site	N	$a_q$	$b_q$	$c_q$	$d_q$	$e_q$	Stability	Stability range
CS-VF0	2626	1.37	0.43	− 1	− 0.51	0	Unstable	$-3.3< z/\varLambda \le -0.001$
	2123	–	–	–	–	2.58	Stable	$1.7\times 10^{-3} \le z/\varLambda \le$ 1.53
CS-SF8	1220	1.1	0.03	− 1	− 0.25	0	Unstable	$-7.59 < z/\varLambda \le -9.7\times 10^{-4}$
	654	–	–	–	–	3.12	Stable	$6.1\times 10^{-4} \le z/\varLambda \le$ 4.38
CS-SF1	778	1.11	0.08	− 1	− 0.33	0	Unstable	$-2.71 < z/\varLambda \le -3.7\times 10^{-3}$
	1268	–	–	–	–	5.25	Stable	$2.5\times 10^{-3}\le z/\varLambda \le$ 1.84
CS-NF10	1092	1.07	0.26	− 1	− 0.36	0	Unstable	$-5.28 < z/\varLambda \le -3\times 10^{-3}$
	2319	–	–	–	–	3.83	Stable	$2.2\times 10^{-3} \le z/\varLambda \le$ 4.98
CS-NF27	364	1.18	0.04	− 1	− 0.27	0	Unstable	$-1.82 < z/\varLambda \le -4.6\times 10^{-3}$
	2166	–	–	–	–	5.28	Stable	$3.2\times 10^{-3} \le z/\varLambda \le$ 1.39
Reference (Cabauw)	2919	0.99	$3.1\times 10^{-2}$	− 1	− 0.288	0	Unstable	$-2.87 < z/\varLambda \le -7\times 10^{-5}$
	5203	–	–	–	–	2.74	Stable	$1.4\times 10^{-5} \le z/\varLambda \le$ 5.5

The coefficients $a_{\theta }$ ($a_q$), $b_{\theta }$ ($b_q$), $c_{\theta }$ ($c_q$), $d_{\theta }$ ($d_q$), $e_{\theta }$ ($e_q$) refer to the general relation in Eq. 6 (Eq. 7) and *N* is the number of available data points for every stability range

**Fig. 12:**
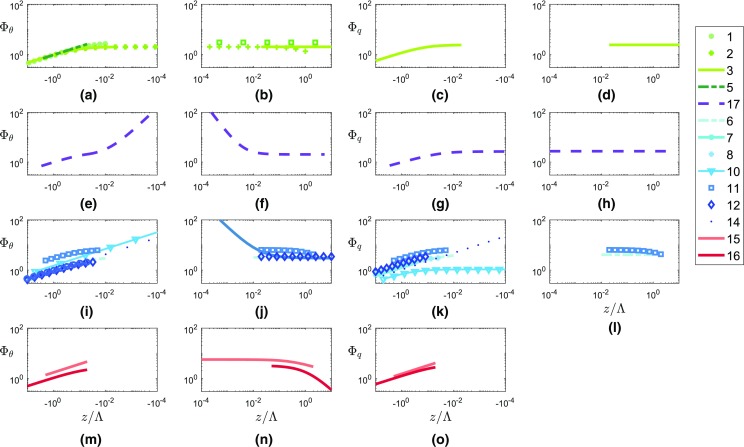
As in Fig. 1, but for different types of terrain: horizontally homogeneous and flat (**a**–**d**), weakly inhomogeneous and flat (**e**–**h**), horizontally inhomogeneous and flat (**i**–**l**) and complex terrain (**m**–**o**). Numbers in the legend refer to Table 1

**Fig. 13:**
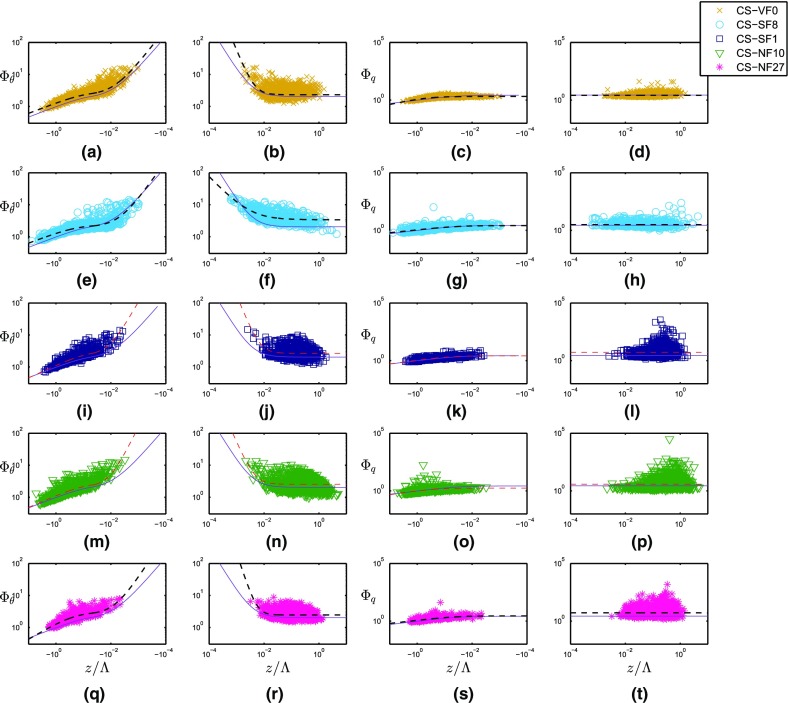
Non-dimensional temperature (left two columns) and humidity (right two columns) standard deviations as a function of local stability for the CS-VF0 (**a**–**d**), CS-SF8 (**e**–**h**), CS-SF1 (**i**–**l**), CS-NF10 (**m**–**p**) and CS-NF27 (**q**–**t**) sites for unstable (left) and stable stratification (right). Data correspond to the first level of every site. Purple solid line: reference curve, black and red dashed lines: best-fit curves

**Fig. 14:**
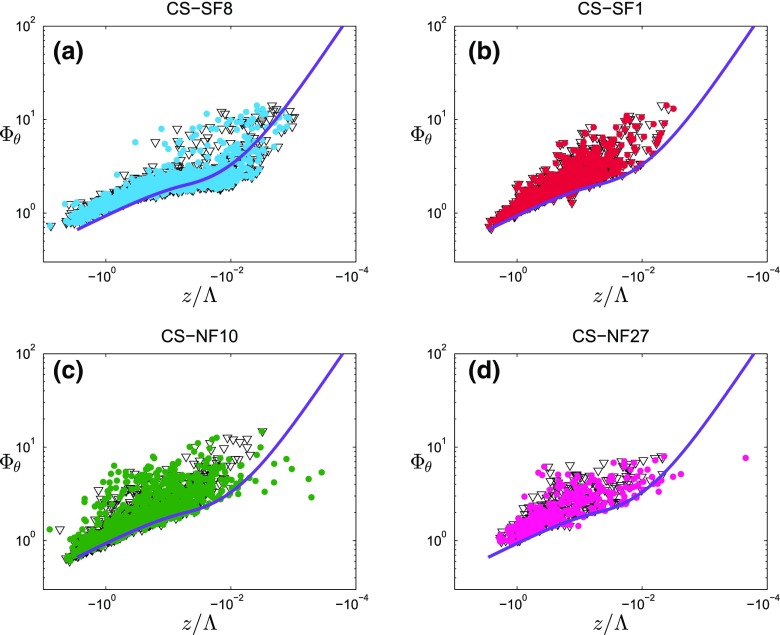
As in Fig. 10 for the CS-SF8 (**a**), CS-SF1 (**b**), CS-NF10 (**c**) and CS-NF27 (**d**) sites. $\triangledown$: slope-normal coordinates, o: vertical coordinates

## Appendix

The Appendix shows several figures in more detail. Also, Table 6 compiles the detailed parameters for the obtained similarity functions and stability ranges for each of the i-Box sites. In Fig. 12, the similarity functions for $\varPhi _{\theta }$ and $\varPhi _q$ from the literature review are shown separately for HHF, WIF, HIF and complex terrain, similar to Fig. 1. Every type of terrain is depicted with a different colour group: HHF terrain curves are shown with green colours, WIF terrain with yellow, HIF terrain with blue and complex terrain with red colours. Figure 13 shows $\varPhi _{\theta }$ and $\varPhi _q$ as a function of $z/\varLambda$ similar to Fig. 7, but separately for every i-Box site.

In Fig. 14, $\varPhi _{\theta }$ is plotted as a function of $z/\varLambda$, with slope-normal and vertical coordinates for the four i-Box sites with sloping terrain (the CS-SF8, CS-SF1, CS-NF10 and CS-NF27 sites), similar to Fig. 10.
